# Solar-driven highly selective conversion of glycerol to dihydroxyacetone using surface atom engineered BiVO_4_ photoanodes

**DOI:** 10.1038/s41467-024-49662-7

**Published:** 2024-06-28

**Authors:** Yuan Lu, Byoung Guan Lee, Cheng Lin, Tae-Kyung Liu, Zhipeng Wang, Jiaming Miao, Sang Ho Oh, Ki Chul Kim, Kan Zhang, Jong Hyeok Park

**Affiliations:** 1 School of Materials Science and Engineering, Nanjing University of Science and Technology, Nanjing, China; 2 Department of Chemical and Biomolecular Engineering, Yonsei University, 50 Yonsei-ro, Seodaemun-gu, Seoul, Republic of Korea; 3 Computational Materials Design Laboratory, Department of Chemical Engineering, Konkuk University, Seoul, the Republic of Korea; 4 Department of Energy Science, Sungkyunkwan University, Suwon, Republic of Korea; 5 Department of Energy Engineering, Institute for Energy Materials and Devices, Korea Institute of Energy Technology (KENTECH), Naju, Republic of Korea

**Keywords:** Electrochemistry, Catalysis, Photocatalysis

## Abstract

Dihydroxyacetone is the most desired product in glycerol oxidation reaction because of its highest added value and large market demand among all possible oxidation products. However, selectively oxidative secondary hydroxyl groups of glycerol for highly efficient dihydroxyacetone production still poses a challenge. In this study, we engineer the surface of BiVO_4_ by introducing bismuth-rich domains and oxygen vacancies (Bi-rich BiVO_4-x_) to systematically modulate the surface adsorption of secondary hydroxyl groups and enhance photo-induced charge separation for photoelectrochemical glycerol oxidation into dihydroxyacetone conversion. As a result, the Bi-rich BiVO_4-x_ increases the glycerol oxidation photocurrent density of BiVO_4_ from 1.42 to 4.26 mA cm^−2^ at 1.23 V vs. reversible hydrogen electrode under AM 1.5 G illumination, as well as the dihydroxyacetone selectivity from 54.0% to 80.3%, finally achieving a dihydroxyacetone production rate of 361.9 mmol m^−2^ h^−1^ that outperforms all reported values. The surface atom customization opens a way to regulate the solar-driven organic transformation pathway toward a carbon chain-balanced product.

## Abstract (web summary)

Photoelectrochemical oxidation of glycerol to produce dihydroxyacetone is limited by its low selectivity. Here, a bismuth vanadate photoanode enriched with a bismuth-rich surface and containing oxygen vacancies was utilized to overcome this dilemma.

## Introduction

Glycerol oxidation reaction (GOR) has garnered significant attention in recent years, due to its economic advantages stemming from low raw material costs (~ US $0.11 per kg) and the potential to yield a diverse range of products^1–4^. However, previously reported high-efficiency electro-oxidation methods often lead to the breaking of the C-C bond of glycerol, tending to the formation of low-carbon products, such as formic acid (FA)^5–7^. Despite the relatively modest market price of FA (approximately US $0.4 per kg), which places a constraint on the overall potential for value enhancement of GOR^8,9^. The prevalence of primary hydroxyl oxidation in glycerol primarily gives rise to the phenomenon of producing products, featuring asymmetric and delicate functional groups. In contrast, dihydroxyacetone (DHA), originating from secondary hydroxyl oxidation, yields a three-carbon product with a robust carbon chain. DHA is particularly attractive, driven by substantial market demand and a high market value (approximately US $150 per kg), especially within the cosmetic industry, establishing it as the most coveted product within the GOR^10,11^. As a result, the pursuit of maintaining a balanced carbon chain to selectively produce DHA becomes an appealing endeavor.

Bismuth vanadate (BiVO_4_) is considered as one of the most potential photoanode materials in photoelectrochemical (PEC) cells because of its suitable energy band position and large light absorption waveband^12,13^. It is essential to highlight that the primary product resulting from glycerol oxidation over the BiVO_4_ photoanode is DHA. However, the modest 50% selectivity of DHA products for BiVO_4_ proves to be below expectations, leading to inefficient solar conversion in the GOR process^14^. While various strategies, such as optical absorption and charge separation regulation, have been implemented to enhance solar-driven GOR conversion for DHA production^15,16^, achieving an improvement in DHA selectivity remains challenging. Recent observations indicate that the bismuth (Bi) atom exhibits a heightened electrostatic adsorption capacity for the secondary hydroxyl of glycerol. This insight suggests that the origin of DHA selectivity is likely associated with the surface Bi atoms of BiVO_4_^17–19^. Nevertheless, the intrinsic distribution of Bi atoms on the BiVO_4_ surface does not exert a decisive influence on directing the GOR reaction pathway due to the atomic structure of monoclinic BiVO_4_, which appears as layered stacking. In this structure, the VO_4_ unit cell adjacent to the cation restricts the contact of Bi atoms with the electrolyte^20,21^. Hence, the prospect of exposing a greater fraction of Bi atoms on the surface of the BiVO_4_ photoanode shows significant potential for achieving highly selective DHA production.

Herein, a photoanode film composed of surface Bi-rich BiVO_4_ particle with mainly exposed the (010) facet is synthesized using a straightforward alkaline immersion method. The Bi-rich BiVO_4_ exhibits an elevated surface potential along with notably amplified secondary hydroxyl adsorption for glycerol. Furthermore, additional oxygen vacancies (O_v_) are introduced to enhance the interaction frequency of Bi atoms at the interface, while leading to an improvement in surface charge transport efficiency. Consequently, the photocurrent density of Bi-rich BiVO_4-x_ photoanode in GOR increases from 1.42 to 4.26 mA cm^−2^ at 1.23 V vs. RHE under AM 1.5 G illumination, accompanied by a selectivity increase of DHA product from 54% to 80.3%, finally achieving a DHA conversion of 361.9 mmol m^−2^ h^−1^ that is the highest value so far.

## Results

**Fig. 1:**
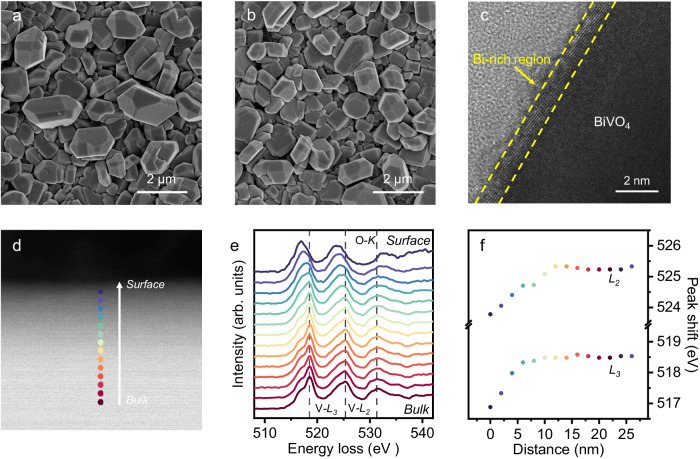
Bi-rich surface construction of BiVO_4_ photoanode. **a**, **b** Top-view SEM image of Bi-rich BiVO_4_ and Bi-rich BiVO_4-x_. **c** HR-TEM image of Bi-rich BiVO_4-x_. **d** STEM image of Bi-rich BiVO_4-x_ particle with the probing path shown by the dotted line, scale bar: 2 nm, (**e**) the corresponding EELS spectrum of Bi-rich BiVO_4-x_. **f** the peak shift of V-*L*_2,3_ edge with depth.

BiVO_4_ photoanodes composed of (010) crystal plane exposed micron-sized BiVO_4_ particles were synthesized by a seed-assisted hydrothermal reaction^22^, as determined by field emission scanning electron microscopy (FE-SEM) images in Supplementary Figs. 1–3. The generation of a Bi-enriched surface and the introduction of O_v_ on the surface of BiVO_4_ were accomplished through alkali solution etching, followed by sequential electrochemical reduction. Compared to bare BiVO_4_, both Bi-rich BiVO_4_ and Bi-rich BiVO_4-x_ have negligible changes in their morphologies (Fig. 1a, b) and crystal structures (Supplementary Fig. 4). Nevertheless, the inductively coupled plasma-mass spectrometry (ICP-MS) results in Supplementary Fig. 5 shows the V atoms being leached from the BiVO_4_ after alkali solution treatment. To understand where the V in solution after alkali etching comes from, Raman spectra of BiVO_4_, Bi-rich BiVO_4,_ and Bi-rich BiVO_4-x_ are compared, since the Raman peaks of BiVO_4_ can be associated to the stretching and vibration of the V-O bond^23^. As shown in Supplementary Fig. 6, the almost unchanged Raman peaks imply that the loss of V after alkali etching does not form V vacancies in BiVO_4_ crystal, rather which might be mainly originating from surface VO_4_^3-^ loss^24^. Correspondingly, high-resolution transmission electron microscopy (HR-TEM) images of the Bi-rich BiVO_4-x_ in Fig. 1c and Supplementary Figs. 7, 8 demonstrate obvious contrast change near the edge and bulk, while element mapping image shows that Bi, V, and O elements are evenly distributed in the grains of Bi-rich BiVO_4-x_, indicating the V loss from the surface shallow region (Supplementary Fig. 9).

To further determine the possible surface component change, electron energy-loss spectroscopy (EELS) was employed. The EELS spectra of BiVO_4_, Bi-rich BiVO_4,_ and Bi-rich BiVO_4-x_ were extracted linearly from the surface to the bulk region with a 26 nm depth, and the transitions in the fine structure of the vanadium *L*_2,3_ edge (V-*L*_2,3_) and the O-K edge at 14 sequential points from the surface to the bulk are shown in Fig. 1d, Supplementary Figs. 10, 11. A high-energy shift of the V-*L*_2,3_, which is caused by V^5+^ reducing more than two valence states^25,26^, can be observed from the surface to the bulk of BiVO_4_ (Fig. 1e). The energy changes of the vanadium *L*_2,3_ edge against depth is plotted in Fig. 1f, Supplementary Figs. 10, 11, accordingly. A remarkable V-*L*_2,3_ peak shift in the region about 10 nm deep for Bi-rich BiVO_4_ and Bi-rich BiVO_4-x_ can be ascribed to V atoms loss. However, comparison of O-*K* edge of Bi-rich BiVO_4_ and Bi-rich BiVO_4-x_, the O_v_ in Bi-rich BiVO_4-x_ leads to a more obvious low-energy shift, which is consistent with the previous report^27^. X-ray photoelectron spectroscopy (XPS) spectra further provide reasonable evidence for the absence of V atoms and the formation of O_v_ (Supplementary Fig. 12), where the blue shift of the V 2p peak and the increase of the O_v_ peak are lined with the above.

**Fig. 2:**
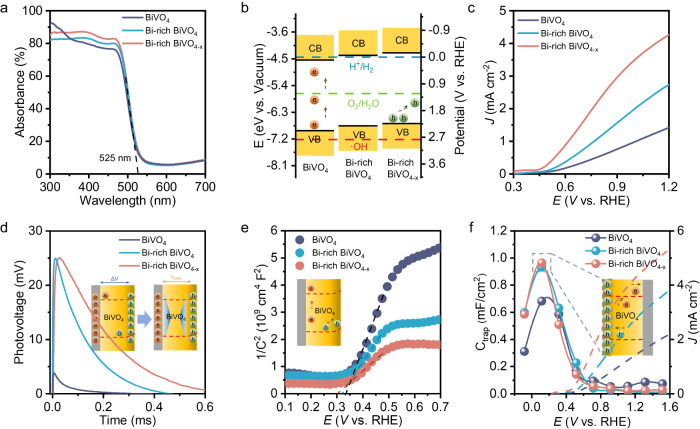
PEC GOR performance and charge behavior characterization. **a** UV–vis absorption spectra of BiVO_4_, Bi-rich BiVO_4_ and Bi-rich BiVO_4-x_ photoanodes. **b** The bond alignments of BiVO_4_, Bi-rich BiVO_4_ and Bi-rich BiVO_4-x_ photoanodes. **c** *J-V* curves of BiVO_4_, Bi-rich BiVO_4_ and Bi-rich BiVO_4-x_ photoanodes in 0.5 M Na_2_SO_4_ (pH = 2) with 0.1 M glycerol under AM 1.5 G illumination. **d** The TS-SPV responses of BiVO_4_, Bi-rich BiVO_4_ and Bi-rich BiVO_4-x_ photoanodes, and (**e**) Mott−Schottky plots measured under AM 1.5 G illumination conditions. **f** *J–V* curves (dashed lines) and C_trap_ values (solid lines and dots) obtained on BiVO_4_, Bi-rich BiVO_4_ and Bi-rich BiVO_4-x_ photoanodes.

UV-vis absorption spectra of BiVO_4_, Bi-rich BiVO_4_ and Bi-rich BiVO_4-x_ show a similar absorption edge at 525 nm, whereas both Bi-rich surface and O_v_ improve the light absorption ability to some extent (Fig. 2a). It is noting that the UV-vis absorption curve of bare BiVO_4_ display two clear shoulders, which are ascribed to the charge transfer transition involving the V-O component and Bi and V centers^28^. Remarkably, the first shoulder (around 300–350 nm) of the Bi-rich BiVO_4_ and Bi-rich BiVO_4-x_ almost disappears, probably, because the charge-transfer transition centered at V is weakened due to the construction of the Bi-rich surface^23^. The band positions of all photoanodes are established by their Tauc plots and valence band (VB)-XPS (Supplementary Figs. 13, 14), and the band structure diagrams are displayed in Fig. 2b. Both the Bi-rich BiVO_4_ and Bi-rich BiVO_4-x_ present a slight shift toward the vacuum level relative to the BiVO_4_. Under the premise that the VB position is appropriate, the upward shift of the band level is generally beneficial to achieve a more favorable band bending at the solid/liquid interface for efficient electron–hole separation^29,30^.

Figure 2c and Supplementary Figs. 15–18 show the linear sweep voltammetry (LSV) curves of BiVO_4_, Bi-rich BiVO_4_, Bi-rich BiVO_4-x_ photoanodes at a scanning rate of 20 mV s^−1^ in a 0.5 M Na_2_SO_4_ electrolyte (pH = 2) with 0.1 M glycerol under AM 1.5 G illumination (100 mW cm^−2^). The GOR photocurrent densities of the BiVO_4_, Bi-rich BiVO_4,_ and Bi-rich BiVO_4-x_ are 1.42, 2.74, and 4.26 mA/cm^2^ at 1.23 V vs. RHE, respectively, indicating that both Bi-rich surface and O_v_ can boost the PEC oxidation performance of BiVO_4_. To understand the role of Bi-rich surface and O_v_ in PEC GOR, the charge transport efficiencies and the charge transfer efficiencies were evaluated by measuring their photocurrent densities using a hole scavenger (Supplementary Fig. 19) and calculating the theoretical photocurrent densities (Supplementary Fig. 20)^31,32^. It can be seen that the charge transport efficiency of bare BiVO_4_ is 29.79%, which is increased to 53.48% after the formation of a Bi-rich surface, and further increased to 67.15% by the introduction of O_v_ (Supplementary Fig. 21). Interestingly, for the carrier transfer efficiencies, the effect of Bi-rich surface is negligible, while the presence of O_v_ leads to a substantial increase from the initial 81.97% to 98.86%.

Transient-state surface photovoltage (TS-SPV) response measurements (Fig. 2d) were employed to investigate the photogenerated charge dynamics process. In terms of SPV response, Bi-rich BiVO_4,_ and Bi-rich BiVO_4-x_ exhibit stronger positive signals compared to BiVO_4_, implying more favorable accumulation of photogenerated holes on the surface. Moreover, compared with the Bi-rich BiVO_4_ photoanode, the Bi-rich BiVO_4-x_ favor long-lived holes, which is benefiting for anodic oxidation^33,34^. Kelvin probe force microscopy (KPFM) further demonstrates the different surface potential caused by the Bi-rich surface and O_v_ (Supplementary Figs. 22–24). Notably, the Bi-rich surface exhibits a noticeably brighter appearance compared to the pure counterparts (either BiVO_4_ or BiVO_4-x_). The increased brightness indicates a stronger charge separation-associated high surface potential, aligning with the findings from the TPV results^35^. The varied frequency Mott-Schottky (MS) curves for various photoanodes were analyzed to estimate reliable band edges. The band edge positions derived from these curves are consistent across different frequencies, indicating their frequency independence (Supplementary Figs. 25, 26). For a clearer comparison, Fig. 2e displays the MS curves of BiVO_4_, Bi-rich BiVO_4_, and Bi-rich BiVO_4-x_ measured at 4000 Hz. As a result, both Bi-rich BiVO_4_ and Bi-rich BiVO_4-x_ exhibit a lower slope compared to BiVO_4_, suggesting a higher donor density. In addition, the onset of MS plots for the Bi-rich BiVO_4_ and Bi-rich BiVO_4-x_ photoanodes demonstrated a gradual cathodic shift, suggesting a greater band bending^36,37^. The cathodic shift of flat band potential is associated with the improved surface charge separation ability, which could imply the accumulation of photogenerated holes within the surface capacitive layer^38,39^. The photocurrent density and fitted capacitance of surface states (C_trap_) calculated to prove this conclusion (Supplementary Fig. 27). As shown in Fig. 2f, the C_trap_ values for Bi-rich BiVO_4_ and Bi-rich BiVO_4-x_ photoanodes are larger than that for BiVO_4_ photoanode at the applied bias smaller than onset potential, which further indicates the holes accumulation in the surface capacitance layer. When the applied bias exceeds onset potential, the C_trap_ values of Bi-rich BiVO_4_ and Bi-rich BiVO_4-x_ photoanodes are faster decreased than BiVO_4_ photoanode, which can be ascribed to that a large number of accumulated charges are released rapidly and participate in the glycerol oxidation reaction, resulting in higher photocurrent density^40,41^. Overall, the Bi-rich surface creates a capacitive layer that is more conducive to the accumulation of photogenerated holes and stores sufficient charges for the subsequent oxidation reaction, which explains the better performance of the photoanodes of Bi-rich BiVO_4-x_.

**Fig. 3:**
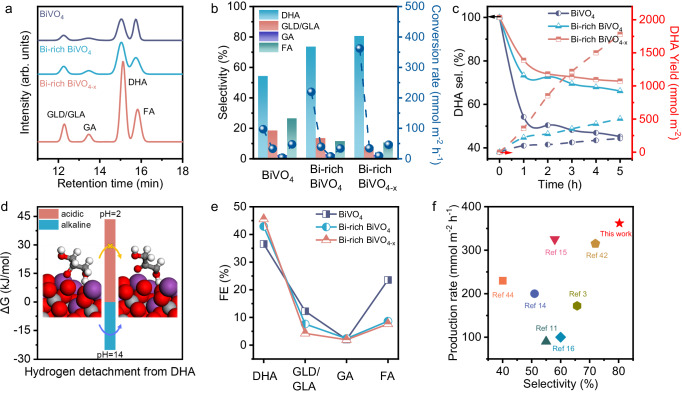
Analysis of the product selectivity and conversion rate in PEC GOR. **a** HPLC spectra of the products and (**b**) conversion rate and selectivity of the main liquid products using the BiVO_4_, Bi-rich BiVO_4,_ and Bi-rich BiVO_4-x_ photoanodes under AM 1.5 G illumination at 1.23 V vs. RHE. **c** DHA selectivity and yields vs. reaction time under AM 1.5 G illumination at 1.23 V vs. RHE. **d** The change in the DFT-calculated Gibbs free energy associated with the initial hydrogen detachment for the decomposition of the DHA adsorbed on the Bi-rich BiVO_4-x_ surface at two different pH conditions: pH = 2 (acidic) and pH = 14 (alkaline). **e** Faradaic efficiency of different products on BiVO_4_, Bi-rich BiVO_4_ and Bi-rich BiVO_4-x_ photoanodes. **f** Summary of the DHA selectivity and conversion rate of PEC GLY oxidation by various photoanodes published in recent years.

The products from PEC GOR were quantitatively analyzed by high-performance liquid chromatography (HPLC). Similar to previous reports^14–16^, the main products of GOR using BiVO_4_ as photoanode include dihydroxyacetone (DHA), glyceric acid (GLA), glyceraldehyde (GLD), glycolic acid (GA) and FA (Fig. 3a, Supplementary Fig. 28). The amount of glycerol can be determined by Nuclear Magnetic Resonance (NMR) analysis (Supplementary Fig. 29). The incorporation of the Bi-rich surface notably enhances the peak height of DHA, suggesting that the DHA emerges as the predominant product. All peak signals are witnessed further enhancement upon the introduction of O_v_, indicating an improved conversion of GOR. The Bi-rich level and oxygen vacancy concentration on the surface of BiVO_4_ photoanodes is adjusted through control of their alkali soaking time and electroreduction duration to find the optimal conditions for DHA production (Supplementary Figs. 30, 31). The Bi-rich BiVO_4_ and Bi-rich BiVO_4-x_ photoanodes constructed under these conditions serve as the subjects of study in the following sections. The specific selectivity and conversion after one-hour reaction for BiVO_4_, Bi-rich BiVO_4_, BiVO_4-x_ and Bi-rich BiVO_4-x_ photoanodes are shown in Fig. 3b. It can be seen that the selectivity of DHA product is increased from 54.0% of BiVO_4_ to 73.3% of Bi-rich BiVO_4_, then to 80.3% of Bi-rich BiVO_4-x_. The further enhanced selectivity of DHA for Bi-rich BiVO_4-x_ might be originating from more Bi atom exposure due to the formation of O_v_ (Supplementary Fig. 32). Correspondingly, the DHA production rate is elevated from 96.8 mmol m^−2^ h^−1^ of BiVO_4_ to 219.2 mmol m^−2^ h^−1^ of Bi-rich BiVO_4_, then to 361.9 mmol m^−2^ h^−1^ of Bi-rich BiVO_4-x_. To understand the stability of GOR, the reaction time was extended to 5 h (Supplementary Fig. 33), and the selectivity and yield of the DHA are shown in Fig. 3c. As the reaction time increases, the selectivity of all photoanodes towards DHA experiences a certain degree of fading due to the occurrence of peroxidation reactions^42^. Finally, the selectivity of the Bi-rich BiVO_4-x_ photoanode towards DHA tends to be stabilized after 5 h of reaction time, consistently maintaining a commendable performance level of 70.7%. Etching XPS was utilized to analyze the changes in the surface elemental concentration distribution of Bi-rich BiVO_4-x_ photoanodes before and after 5 h GOR (Supplementary Fig. 34). As shown in Supplementary Fig. 35, the photoanode still maintained a surface Bi-rich state and abundant in O_v_ after GOR, demonstrating its structural stability during prolonged working.

To explore the reason for the relatively stable accumulation of DHA in the reaction system, spin-polarized density functional theory (DFT) was employed to calculate the desorption preference of DHA at the active sites (Supplementary Fig. 36 and Supplementary Table 1). The calculated value is negative (− 17.49 kJ/mol), indicating that DHA is not inclined to remain continuously adsorbed on the active sites for further overoxidation during the reaction process (Supplementary Fig. 37). The oxidation selectivity of glycerol substrate and DHA product is further demonstrated by PEC glycerol/DHA mixture oxidation, where the molar ratios of glycerol/DHA mixtures are established to be 10:1, 1:1 and 1:10, respectively (Supplementary Fig. 38). It can be seen that the photocurrent density of PEC glycerol/DHA mixture oxidation appears a drop compared to the photocurrent density of PEC GOR only when the molar ratio of glycerol/DHA mixture is reduced to 1:10. Further analysis of products reveals that the amount of DHA produced is almost same in the glycerol/DHA mixtures (Supplementary Figs. 39,  40). Therefore, the dropped photocurrent density in the low molar ratio of glycerol/DHA mixture might be ascribed to the factor of glycerol mass transfer, not that DHA is oxidized. Moreover, because the atomic spacing between two Bi atoms is about 3.09 Å (Supplementary Fig. 8), nearly twice that of a C-C single bond (1.54 Å). The spacing makes it difficult for neighboring carbon atoms to be adsorbed simultaneously, which may be a cause of the suppression of C-C bond breaking^43^. The above two reasons would act as main factor for continuous DHA production.

In addition, the effect of acidic and alkaline environments on its preference for carbon chain dehydrogenation (a key step in C-C bond breaking) was also noted. The calculation results indicate that the Gibbs free energy change for DHA dehydrogenation is 43.44 kJ/mol in acidic conditions, a positive value, while in alkaline conditions, it is − 25.06 kJ/mol, a negative value (Fig. 3d and Supplementary Table 2). This demonstrates that the carbon chain dehydrogenation process of DHA is thermodynamically unfavorable under acidic conditions, which is consistent with the experimental results (Supplementary Fig. 41). Therefore, the low pH electrolyte we use similarly contributes to the stabilization of the carbon chain of DHA. The product selectivity and yield of Bi-rich BiVO_4-x_ photoanode at different potentials are shown in Supplementary Fig. 42. Obviously, the product selectivity at different potentials is stable, but the conversion rate obviously increases with the increase of potential. This improvement is attributed to the larger photocurrent density at higher potentials. The Faradaic efficiency (FE) of each photoanode for different products are calculated and shown in Fig. 3e. The shortfall in the total FE was proven to be due to the production of oxygen (Supplementary Fig. 43). The FE of the products resulting from the primary hydroxyl oxidation reaction pathway (GLD/GLA, GA, FA) exhibited a decrease for the Bi-rich surface, while increasing the FE of DHA. The change in FE provides favorable evidence of a shift in the glycerol oxidation reaction pathway^44^. The PEC glycerol oxidative DHA performances in terms of selectivity and production rate are compared in Fig. 3f, demonstrating the best performances among all reported works.

**Fig. 4:**
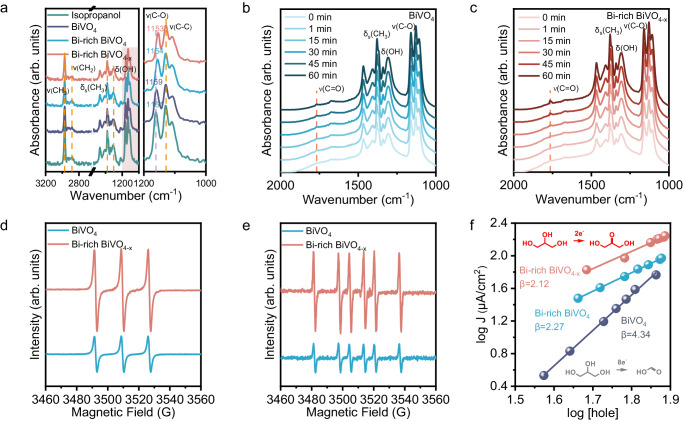
Verification of glycerol adsorption on the photoanode surface and investigation into the glycerol oxidation mechanism. **a** FT-IR spectra of isopropanol on BiVO_4_, Bi-rich BiVO_4_ and Bi-rich BiVO_4-x_ photoanodes. **b**, **c** In situ FT-IR spectra of the dynamic oxidation process of isopropanol on BiVO_4_, Bi-rich BiVO_4,_ and Bi-rich BiVO_4-x_ photoanodes under AM 1.5 G illumination for 60 min. **d**, **e** EPR detection of photogenerated holes and carbon-centered radicals over illuminated BiVO_4_ and Bi-rich BiVO_4-x_ photoanodes. **f** Relationship between the photocurrent density and surface-hole density.

To understand the role of Bi-rich surface in PEC GOR, in-situ Fourier transform infrared (FT-IR) spectra of BiVO_4_, Bi-rich BiVO_4,_ and Bi-rich BiVO_4-x_ photoanode are provided to investigate the changes in the adsorption state of glycerol. For comparison purposes, isopropanol and propanol with secondary and primary hydroxyl respectively, are employed as reagents. As shown in Fig. 4a, when isopropanol was adsorbed on the surfaces of BiVO_4_, Bi-rich BiVO_4,_ and Bi-rich BiVO_4-x_ photoanode, the peaks of ν(C-O) band all shift to low wavenumbers. Notably, either BiVO_4_ or BiVO_4-x_ with Bi-rich surfaces exhibits more pronounced shifts (from 1161 to 1154 and 1153 cm^−1^ respectively). The spectral alteration can be attributed to the bond breaking caused by the adsorption of the hydroxyl group of isopropanol to the surface exposed Bi atom, implying that Bi-rich surface is more conducive to the adsorption of secondary hydroxyl groups of glycerol^45,46^. In contrast, the adsorption of propanol did not cause any visible peak shift of each sample, which indicates that the primary hydroxyl groups of glycerol would be randomly oxidized on the surface of BiVO_4_, regardless of surface exposed atoms (Supplementary Fig. 44). Furthermore, in situ FT-IR was performed to analyze the adsorption process of isopropanol on BiVO_4_ and Bi-rich BiVO_4-x_ photoanodes under AM 1.5 G illumination. As shown in Fig. 4b, c, a new characteristic peak is formed at 1765 cm^−1^, attributing to the formation of the carbonyl group (C = O). The appearance of the carbonyl group implies the oxidation of isopropanol to acetone. The C = O signal detected on the surface of the Bi-rich BiVO_4-x_ photoanode exhibits significantly greater intensity than that observed on the BiVO_4_ photoanode over the course of time^42^. The more rapidly increasing signal intensity indicates that aldehyde products accumulate at a faster rate on the Bi-rich BiVO_4-x_ photoanode surface, which is consistent with the superior DHA selectivity of Bi-rich BiVO_4-x_ mentioned above. In addition, the signal of the O = C-O bond cannot be observed in the FT-IR spectra of either BiVO_4_ or Bi-rich BiVO_4-x_ after one hour of illumination. The result was interpreted as the ketone products generated being able to remain in the system for an extended period, which corresponds to the DFT calculation results regarding DHA molecule desorption preference and carbon chain stability discussed above^3^.

Room temperature electron spin resonance (ESR) spectroscopy was investigated to study the main intermediates in the PEC GOR process. As shown in Supplementary Fig. 45, in the absence of H_2_O_2_, production of ·OH by both the BiVO_4_ and Bi-rich BiVO_4-x_ photoanodes is negligible, which is consistent with the previous report^47^. Nevertheless, Bi-rich BiVO_4-x_ exhibits a stronger photogenerated hole signal in comparison to BiVO_4_, which is indicative of GOR occurring under the influence of photogenerated holes (Fig. 4d). While the signal intensity associated with carbon-centered radical is also enhanced to the same extent in the presence of glycerol (Fig. 4e)^48,49^. To further elucidate the reaction mechanism, the rate of glycerol oxidation by surface-trapped holes was determined by analyzing electrochemical impedance spectra (EIS) under varying light intensities (Supplementary Fig. 46). The EIS spectra measured under different light intensities were fitted by equivalent model circuit and electrochemically active surface areas (Supplementary Figs. 47–49). The fitting results are shown in Supplementary Tables 3–5. The log/log plots of the photocurrent density and hole density are displayed in Fig. 4f, and the reaction orders of BiVO_4_, Bi-rich BiVO_4,_ and Bi-rich BiVO_4-x_ photoanodes can be established to be 4.34, 2.27, and 2.12 respectively. The different reaction orders suggest different reaction pathways. As the Bi-rich BiVO_4_ and Bi-rich BiVO_4-x_ photoanodes incline to oxidize the glycerol to DHA via a 2-electron transfer process (Supplementary Table 6), whereas the higher reaction order of BiVO_4_ is due to the fact that its glycerol oxidation product contains more FA which is 8-electron transfer process^50^. The charge-transfer-related tendency exhibited by reaction order corresponds to the hole oxidation mechanism mentioned above^51^.

**Fig. 5:**
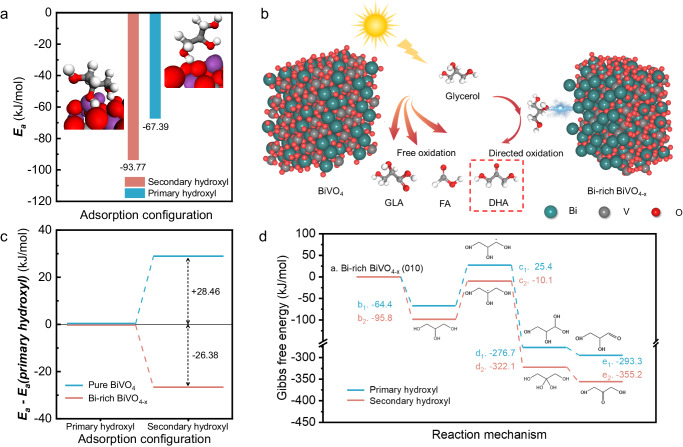
Validation of experimental results through theoretical calculations. **a** The DFT-calculated energies related to glycerol adsorption on the Bi-rich BiVO_4-x_ surface through either the primary or secondary hydroxyl group. **b** Schematic illustration of the PEC glycerol oxidation to DHA using Bi-rich BiVO_4-x_ photoanode. **c** The adsorption energy involving secondary hydroxyl groups in relation to the adsorption energy associated with primary hydroxyl groups for glycerol adsorbed on both pure BiVO_4_ and Bi-rich BiVO_4-x_ surfaces. **d** The Gibbs free energy profiles linked to oxidation processes involving primary and secondary hydroxyl groups on Bi-rich BiVO_4-x_ surfaces.

To further validate the adsorption behavior of secondary hydroxyl of glycerol on the Bi-rich surface, DFT calculations were conducted (Supplementary Fig. 50). The (010) facet of Bi-rich BiVO_4-x_ served as the slab surface model for investigating adsorption energies and oxidation mechanisms of glycerol, the primary hydroxyl and secondary hydroxyl groups were individually adsorbed on the surface-exposed Bi atoms. As shown in Fig. 5a, the adsorption energy for the secondary hydroxyl group of glycerol is found to be − 97.33 kJ/mol, notably lower than − 67.39 kJ/mol predicted for the primary hydroxyl group of glycerol. This observation suggests a higher preference for the adsorption of the secondary hydroxyl group of glycerol on the surface of the Bi-rich BiVO_4-x_ photoanode, making it thermodynamically susceptible to oxidization to DHA (Fig. 5b)^52,53^. Furthermore, Fig. 5c shows the disparity in the adsorption energies of the secondary and primary hydroxyl groups of glycerol on the surfaces of pure BiVO_4_ and Bi-rich BiVO_4-x_, respectively. The adsorption capacity for primary hydroxyl groups of glycerol is observed to be even stronger than that for secondary hydroxyl groups on the (010) facet of BiVO_4_. Upon the removal of V and O atoms from the surface, there is a rapid increase in the adsorption strength for secondary hydroxyl groups. The observation further underscores the substantial impact of exposed Bi atoms on the adsorption behavior of the secondary hydroxyl group of glycerol. Finally, the Gibbs free energy profiles of the oxidation pathways for both primary and secondary hydroxyl groups of glycerol on Bi-rich BiVO_4-x_ surface are shown in Fig. 5d. Evidently, Bi-rich BiVO_4-x_ demonstrates thermodynamic favorability for each step in the oxidation pathway of secondary hydroxyl groups of glycerol compared to primary hydroxyl groups. The free energy difference of 61.9 kJ/mol throughout the entire reaction between the two pathways undeniably designates DHA as the more favorable reaction product.

## Discussion

In this work, a surface component tailoring approach was demonstrated to selectively oxidize glycerol to high value-added DHA. The Bi-rich and O_v_ co-existed surface of BiVO_4_ enabled the photocurrent density of GOR being improved from 1.42 to 4.26 mA cm^−2^ at 1.23 V vs. RHE under AM 1.5 G illumination, while increasing the selectivity of DHA product from 54.0% to 80.3%, finally achieving a DHA production rate of 361.9 mmol m^−2^ h^−1^, marking the highest reported value to date. Comprehensive experimental detection and theoretical calculation confirm the strong electrostatic adsorption of glycerol secondary hydroxyl groups on the Bi-rich surface, bringing about a directional GOR pathway toward DHA via 2-electron transfer process. Meanwhile, the elevated surface potential engendered by the Bi-rich surface and the potent surface charge transfer facilitated by the oxygen vacancies provide favorable reaction dynamics for GOR. This work is expected to provide a scheme through surface atom tailoring instead of co-catalyst introduction to achieve a high-valued carbon chain-balanced product.

## Methods

### Synthesis of BiVO_4_ seed layer on FTO substrates

The BiVO_4_ seed layer was applied by spin-coating the precursor solution onto pristine FTO substrates. To prepare the precursor solution for the BiVO_4_ seed layer, 0.3234 g of Bi (NO_3_)_3_·5H_2_O (Sigma-Aldrich, purity > 99.99%) was dissolved in 1 ml of concentrated HNO_3_ (PFP, 60 wt%), followed by the addition of 2 ml of Milli-Q water. Subsequently, 0.078 g of NH_4_VO_3_ (Sigma-Aldrich, purity ≥ 99%) and 0.167 g of polyvinyl alcohol (PVA, Sigma-Aldrich, purity ≥ 99%) were dissolved in the aforementioned solution and vigorously stirred until it achieved transparency. The precursor solution was then spin-coated onto the FTO substrate at 2500 rpm for 20 s, followed by calcination at 450 °C for 2 h in an air environment.

### Preparation of BiVO_4_ photoanodes

0.1164 g of Bi (NO_3_)_3_·5H_2_O and 0.028 g of NH_4_VO_3_ were dissolved in 1.6 mL of concentrated HNO_3_ (60 wt%). Milli-Q water was added until the total volume reached 60 mL. The BiVO_4_ seed layer was immersed in the solution with the seed layer oriented downward. The solution was then transferred to a Teflon-lined autoclave and heated at 180 °C for a duration of 12 h. The resulting BiVO_4_ was subsequently washed with Milli-Q water and subjected to calcination at 450 °C for 2 h in an air atmosphere.

### Preparation of Bi-rich BiVO_4_ photoanodes

To prepare the Bi-rich BiVO_4_ photoanode, start by dissolving 0.4 g of NaOH (Sigma-Aldrich, 97%) in 100 ml of deionized water. Next, extract 40 ml of the prepared solution and immerse the BiVO_4_ photoanode in it for a period ranging from 60 to 200 s. This step is aimed at removing the surface V atoms. After the soaking process, carefully remove the photoanode, rinse it thoroughly with deionized water, and allow it to dry. The resulting Bi-rich BiVO_4_ photoanode used in this study was obtained after soaking for 150 s.

### Preparation of Bi-rich BiVO_4-x_ photoanodes

Oxygen vacancy generation was carried out in a three-electrode photoelectrochemical (PEC) cell, which included a 1 M potassium borate electrolyte (KBi) with a pH of 9.5. In this setup, a platinum sheet served as the counter electrode, while an Ag/AgCl electrode was employed as the reference electrode. The working electrode consisted of the Bi-rich BiVO_4_ photoanode. It was maintained at a potential of − 0.8 V vs. the Reversible Hydrogen Electrode (RHE) for a duration of 300 s to produce the Bi-rich BiVO_4-x_ photoanode. BiVO_4-x_ was also obtained using the same procedure.

### Material characterization

SEM images were captured using a JSM-7610F-Plus field emission scanning electron microscope. High-resolution transmission electron microscopy (HR-TEM) images and atom-level images were acquired using a JEOL JEM-ARM 200 F (NEOARM) transmission electron microscope with Cs-corrected/energy-dispersive X-ray spectroscopy (EDS)/EELS capabilities. To determine the crystalline structures, X-ray diffraction (XRD) analysis was performed with a Siemens D500/5000 diffractometer using a Bragg–Brentano geometry, employing Cu Kα radiation at 40 keV and 40 mA. X-ray photoelectron spectroscopy (XPS) data were obtained using an SESXPS instrument (ESCA2000, VG Microtech, England). UV-vis absorption spectra were recorded using a UV-vis spectrophotometer (Cary5000, Agilent) and obtained in diffuse reflection mode. Raman analyses were conducted using a Horriba Jovin Yvon LabRam Aramis Raman microscope equipped with a 532 nm laser. Inductively coupled plasma-mass spectrometry (ICP-MS) was carried out using an ICP-MS instrument from Agilent (model 7900). Kelvin probe force microscopy (KPFM) was conducted using an Atomic Force Microscope from Park Systems (model NX-10).

### PEC Measurements

The photoelectrochemical (PEC) performance of all photoanodes was evaluated using a three-electrode optical O-ring cell configuration. In this setup, a platinum (Pt) wire served as the counter electrode, and a saturated calomel electrode was used as the reference. For all PEC measurements, a 0.5 M sodium sulfate electrolyte (pH = 2, with or without 0.1 M glycerol) was consistently employed. Data acquisition was conducted using a CHI 660E electrochemical workstation. Illumination was provided by a solar simulator (100 mW/cm², Peccell Technologies, Yokohama, Japan, PEC-L01), with all electrodes being illuminated from the rear side. Linear sweep voltammetry (LSV) experiments were performed by sweeping the potential in the positive direction at a scan rate of 10 mV/s. The potential measured relative to the Ag/AgCl reference electrode was converted to the potential versus the Reversible Hydrogen Electrode (RHE) using the Nernst equation: E (vs. RHE) = E (vs. Ag/AgCl) + 0.0591 × pH + 0.196. Electrochemical impedance spectroscopy (EIS) measurements were taken by applying a sinusoidal AC perturbation of 5 mV across a frequency range from 0.1 Hz to 1 MHz. Mott-Schottky (MS) curves were obtained at a frequency of 1000, 2000, 4000, and 8000 Hz with an amplitude of 10 mV^54^.

During the evaluation of charge transfer efficiency (η_trans_) and charge separation efficiency (η_sep_), a Na_2_SO_3_ electrolyte was used as the hole scavenger. Here, η_sep_ indicates the fraction of photogenerated holes at the electrode/electrolyte interface, whereas η_trans_ denotes the fraction of these holes that reach the photoanode/electrolyte interface and participate in water oxidation.

The detailed calculation process of the absorbed photocurrent density in BiVO_4_ films was as follows:

1 $${{{{{\rm{\eta }}}}}}_{{{{{{\rm{trans}}}}}}}=\frac{{{{{{{\rm{J}}}}}}}_{{{{{{{\rm{H}}}}}}}_{2}{{{{{\rm{O}}}}}}}}{{{{{{{\rm{J}}}}}}}_{{{{{{{\rm{Na}}}}}}}_{2}{{{{{{\rm{SO}}}}}}}_{3}}}$$

2 $${{{{{\rm{\eta }}}}}}_{{{{{{\rm{sep}}}}}}}=\frac{{{{{{\rm{J}}}}}}_{{{{{{{\rm{Na}}}}}}}_{2}{{{{{{\rm{SO}}}}}}}_{3}}}{{{{{{{\rm{J}}}}}}}_{{{{{{\rm{abs}}}}}}}}$$

The single photon energy was calculated from Eq. (3):

3 $${{{{{\rm{E}}}}}}({{{{{\rm{\lambda }}}}}})={{{{{\rm{h}}}}}}\times {{{{{\rm{C}}}}}}/{{{{{\rm{\lambda }}}}}}$$

where E(λ) is the photon energy (J), h is the Planck constant (6.626 × 10^−34^ Js), C is the speed of light (3 × 10^8 ^m s^−1^) and λ is the photon wavelength (nm).

The solar photon flux was then calculated according to Eq. (4):

4 $${{{{{\rm{Flux}}}}}}({{{{{\rm{\lambda }}}}}})={{{{{\rm{P}}}}}}({{{{{\rm{\lambda }}}}}})/{{{{{\rm{E}}}}}}({{{{{\rm{\lambda }}}}}})$$

where Flux(λ) is the solar photon flux (m^−2^ s^−1^ nm^−1^), and P(λ) is the solar power flux (W m^−2^ nm^−1^). The theoretical absorbed photocurrent density under solar illumination (AM 1.5 G), J_abs_ (A m^−2^), was then calculated by integrating the solar photon flux between 300 and 525 nm, as shown in Eq. (6):

5 $${{{{{{\rm{J}}}}}}}_{{{{{{\rm{abs}}}}}}}={{{{{\rm{e}}}}}}\times {\int }_{300}^{{{{{{\rm{X}}}}}}}{{{{{\rm{\eta }}}}}}_{{{{{{\rm{har}}}}}}}{{{{{\rm{Flux}}}}}}({{{{{\rm{\lambda }}}}}}){{{{{\rm{d}}}}}}{{{{{\rm{\lambda }}}}}}$$

where e is the elementary charge (1.602 × 10^−19^ C), η_har_ is the absorption spectrum^55^.

### Photoelectrochemical glycerol oxidation measurements

PEC glycerol (GLY) oxidation measurements were conducted inside a sealed H-type glass cell over a 5-h duration. To separate the anode from the cathode chamber, a Nafion 212 proton exchange membrane was employed. The electrolyte solution, which included 0.1 M GLY, was composed of a 0.5 M Na_2_SO_4_ aqueous solution with the pH adjusted to 2 by adding a 0.5 M H_2_SO_4_ solution. All other experimental conditions were consistent with those utilized for the PEC water oxidation measurements.

To quantitatively analyze the glycerol oxidation products, PEC oxidation was carried out within a sealed H-type cell at a potential of 1.23 V vs. the Reversible Hydrogen Electrode (RHE) for a duration of 5 h. Following the reaction, 1 mL of the solution was withdrawn from the cell and subjected to analysis using high-performance liquid chromatography (HPLC), specifically an Agilent 1260 Infinity system. The HPLC system was equipped with an Aminex HPX-87 H column (Bio-Rad, 300 × 7.8 mm) was employed for analysis. The column was operated at a temperature of 50 °C and eluted with 10 mM aqueous H_2_SO_4_ as the eluent. The eluent was delivered at a flow rate of 0.5 mL/min. The detection wavelength of the DAD detector is 210 nm. The product selectivity and production rate of glycerol oxidation reaction can be obtained from the following formula:

6 $${{{{\rm{Selectivity}}}}}\left({{{{\rm{DHA}}}}}\right)=\frac{{{{{{\rm{n}}}}}}_{{{{{\rm{DHA}}}}}}}{{{{{{\rm{n}}}}}}_{{{{{\rm{all}}}}}}}\times 100\%=\frac{{{{{{\rm{n}}}}}}_{{{{{\rm{DHA}}}}}}}{{{{{{\rm{n}}}}}}_{{{{{\rm{DHA}}}}}}+{{{{{\rm{n}}}}}}_{{{{{\rm{GLA}}}}}}+{{{{{\rm{n}}}}}}_{{{{{\rm{GA}}}}}}+{{{{{\rm{n}}}}}}_{{{{{\rm{FA}}}}}}}\times 100\% \\=\frac{{{{{{\rm{C}}}}}}_{{{{{\rm{DHA}}}}}}}{{{{{{\rm{C}}}}}}_{{{{{\rm{DHA}}}}}}+{{{{{\rm{C}}}}}}_{{{{{\rm{GLA}}}}}}+{{{{{\rm{C}}}}}}_{{{{{\rm{GA}}}}}}+{{{{{\rm{C}}}}}}_{{{{{\rm{FA}}}}}}}\times 100\%$$

where n_GLA_, n_DHA_, n_GA_ and n_FA_ are the yields of DHA, GLA, GA and FA, respectively. C is the product concentration detected by HPLC. The selectivity of other liquid products was also calculated based on the above equation.

7 $${{{{{\rm{Production}}}}}} \, {{{{{\rm{rate}}}}}}\left({{{{\rm{DHA}}}}}\right)=\frac{{{{{{\rm{C}}}}}}_{{{{{\rm{DHA}}}}}}\times {{{{\rm{V}}}}}}{{{{{\rm{t}}}}}\times {{{{\rm{A}}}}}}\times 100\%$$

where V is the volume of the reaction solution, t is the reaction time, and A is the area of the photoanode. The production rate of other liquid products was also calculated based on the above equation.

8 $${{{{{\rm{Faradaic}}}}}} \, {{{{{\rm{efficiency}}}}}} \, \left({{{{\rm{DHA}}}}}\right) 	= \frac{{{{{{\rm{Number}}}}}} \, {{{{{\rm{of}}}}}} \, {{{{{\rm{holes}}}}}} \, {{{{{\rm{to}}}}}} \, {{{{{\rm{oxidize}}}}}} \, {{{{{\rm{GLY}}}}}} \, {{{{{\rm{to}}}}}} \, {{{{{\rm{DHA}}}}}}}{{{{{{\rm{Number}}}}}} \, {{{{{\rm{of}}}}}} \, {{{{{\rm{collected}}}}}} \, {{{{{\rm{photogenerated}}}}}} \,{{{{{\rm{holes}}}}}}}\times 100\%\\ 	=\frac{{{{{{\rm{e}}}}}}_{{{{{\rm{DHA}}}}}}\times {{{{{\rm{n}}}}}}_{{{{{\rm{DHA}}}}}}\times {{{{\rm{N}}}}}}{{{{{\rm{Q}}}}}/{{{{\rm{n}}}}}}=\frac{2\times {{{{{\rm{C}}}}}}_{{{{{\rm{DHA}}}}}}\times {{{{\rm{V}}}}}\times {{{{\rm{N}}}}}}{{{{{\rm{Q}}}}}/{{{{\rm{n}}}}}}$$

where e_DHA_ is the number of holes required to oxidize one glycerol molecule to one DHA molecule, N is Avogadro’s constant, Q is the quantity of electric charge, and n is the elementary charge. The Faradaic efficiency of other liquid products was also calculated based on the above equation (e_DHA_ = 2, e_GLA_ = 4, e_GA_ = 5, e_FA_ = 8).

9 $${{{{{\rm{Faradaic}}}}}} \, {{{{{\rm{efficiency}}}}}} \, \left({{{{\rm{GLA}}}}}\right) 	=\frac{{{{{{\rm{Number}}}}}} \, {{{{{\rm{of}}}}}}\, {{{{{\rm{holes}}}}}} \, {{{{{\rm{to}}}}}} \, {{{{{\rm{oxidize}}}}}} \, {{{{{\rm{GLY}}}}}} \, {{{{{\rm{to}}}}}} \, {{{{{\rm{GLA}}}}}}}{{{{{{\rm{Number}}}}}} \, {{{{{\rm{of}}}}}} \, {{{{{\rm{collected}}}}}} \, {{{{{\rm{photogenerated}}}}}} \, {{{{{\rm{holes}}}}}}}\times 100\%\\ 	=\frac{{{{{{\rm{e}}}}}}_{{{{{\rm{GLA}}}}}}\times {{{{{\rm{n}}}}}}_{{{{{\rm{GLA}}}}}}\times {{{{\rm{N}}}}}}{{{{{\rm{Q}}}}}/{{{{\rm{n}}}}}}=\frac{4\times {{{{{\rm{C}}}}}}_{{{{{\rm{GLA}}}}}}\times {{{{\rm{V}}}}}\times {{{{\rm{N}}}}}}{{{{{\rm{Q}}}}}/{{{{\rm{n}}}}}}$$

10 $${{{{{\rm{Faradaic}}}}}} \, {{{{{\rm{efficiency}}}}}} \, \left({{{{\rm{GA}}}}}\right) 	=\frac{{{{{{\rm{Number}}}}}} \, {{{{{\rm{of}}}}}} \, {{{{{\rm{holes}}}}}} \, {{{{{\rm{to}}}}}} \,{{{{{\rm{oxidize}}}}}} \, {{{{{\rm{GLY}}}}}} \, {{{{{\rm{to}}}}}} \, {{{{{\rm{GA}}}}}}} {{{{{{\rm{Number}}}}}} \, {{{{{\rm{of}}}}}} \,{{{{{\rm{collected}}}}}} \, {{{{{\rm{photogenerated}}}}}} \, {{{{{\rm{hole}}}}}}} \times 100\%\\ 	=\frac{\frac{2}{3}{\times {{{{\rm{e}}}}}}_{{{{{\rm{GA}}}}}}\times {{{{{\rm{n}}}}}}_{{{{{\rm{GA}}}}}}\times {{{{\rm{N}}}}}}{{{{{\rm{Q}}}}}/{{{{\rm{n}}}}}}=\frac{\frac{2}{3}\times 5\times {{{{{\rm{C}}}}}}_{{{{{\rm{GA}}}}}}\times {{{{\rm{V}}}}}\times {{{{\rm{N}}}}}}{{{{{\rm{Q}}}}}/{{{{\rm{n}}}}}}$$

11 $${{{{{\rm{Faradaic}}}}}} \, {{{{{\rm{efficiency}}}}}} \, \left({{{{\rm{FA}}}}}\right) 	=\frac{{{{{{\rm{Number}}}}}} \, {{{{{\rm{of}}}}}} \, {{{{{\rm{holes}}}}}} \, {{{{{\rm{to}}}}}} \, {{{{{\rm{oxidize}}}}}} \, {{{{{\rm{GLY}}}}}} \,{{{{{\rm{to}}}}}} \, {{{{{\rm{FA}}}}}}}{{{{{{\rm{Number}}}}}} \, {{{{{\rm{of}}}}}} \, {{{{{\rm{collected}}}}}} \, {{{{{\rm{photogenerated}}}}}} \, {{{{{\rm{holes}}}}}}}\times 100\%\\ 	=\frac{\frac{1}{3}\times {{{{{\rm{e}}}}}}_{{{{{\rm{FA}}}}}}\times {{{{{\rm{n}}}}}}_{{{{{\rm{FA}}}}}}\times {{{{\rm{N}}}}}}{{{{{\rm{Q}}}}}/{{{{\rm{n}}}}}}= \frac{\frac{1}{3}\times 8\times {{{{{\rm{C}}}}}}_{{{{{\rm{FA}}}}}}\times {{{{\rm{V}}}}}\times {{{{\rm{N}}}}}}{{{{{\rm{Q}}}}}/{{{{\rm{n}}}}}}$$

where e_GLA_ is the number of holes required to oxidize one glycerol molecule to one GLA molecule, e_GA_ is the number of holes required to oxidize one glycerol molecule to two-thirds GA molecule and e_FA_ is the number of holes required to oxidize one glycerol molecule to three FA molecules^14^.

### Calculation method of reaction order

The equivalent model circuit for fitting EIS results is shown in Figure S37. In this model, C_trap_ represents the charges accumulated at surface states, R_trapping_ represents the resistance in surface-hole trapping, and R_ct,trap_ represents the resistance of interfacial charge transfer. The Nyquist plots exhibit two semicircles for this model. The high-frequency semicircle represents the process of hole trapping by surface states (hole accumulation at the surface), while the radius of the low-frequency semicircle reflects the process of interfacial hole transfer to H_2_O.

The density of surface-trapped holes can be calculated by the following equation:

12 $$\left[{{{{\rm{hole}}}}}\right]={{{{{\rm{C}}}}}}_{{{{{\rm{trap}}}}}}\times {{{{{\rm{V}}}}}}_{{{{{\rm{bias}}}}}}\frac{{{{{{\rm{R}}}}}}_{{{{{\rm{ct}}}}},{{{{\rm{trap}}}}}}}{{{{{{\rm{R}}}}}}_{{{{{\rm{s}}}}}}+{{{{{\rm{R}}}}}}_{{{{{\rm{trap}}}}}}+{{{{{\rm{R}}}}}}_{{{{{\rm{ct}}}}},{{{{\rm{trap}}}}}}}/{{{{\rm{S}}}}}$$

where V_bias_ is the applied bias and S is the active area of the electrode.

The reaction rate was represented by the photocurrent density (*J*). The reaction order (*β*) of surface-trapped holes in water oxidation can be obtained by fitting the data.

13 $${{{{{\rm{J}}}}}}={{{{{\rm{k}}}}}} \, {[{{{{{\rm{hole}}}}}}]}^{{{{{\rm{\beta}}}}}}$$

14 $$\log {{{{{\rm{J}}}}}}={{{{{\rm{\beta}}}}}} \,\log ([{{{{{\rm{hole}}}}}}])+\,\log {{{{{\rm{k}}}}}}$$

where k is the rate constant of the reaction, and β is the reaction order^56^.

### Computational methods

All Density functional theory (DFT) calculations were performed using the Vienna ab initio simulation package^57,58^. The projector augmented wave method, along with the Perdew-Burke-Ernzerhof parameterization of the generalized gradient approximation with spin polarization, was employed for the exchange-correlation functional^59,60^. The plane-wave cutoff energy was additionally set to 520 eV. Hubbard U correction with *V* = 2.7 eV was applied to fully describe the strong *d*-electron correlation of transition metal, V^61^. During the geometry optimization of bulk phases, all atoms were fully relaxed with an energy convergence tolerance of 10^−5 ^eV per atom and the final forces were converged to less than 0.02 eV/Å. Monkhorst-Pack grid points with 6 × 3 × 9 *k*-meshes were utilized for optimizing bulk BiVO_4_ phase^62^. The geometrically optimized bulk BiVO_4_ model was cleaved into (010) direction, exposing surfaces with four-coordinated bismuth^24^. Each slab model had 2 × 2 × 1 supercell to prevent interactions of guest molecules, glycerols, in adjacent cells, while keeping a vacuum thickness greater than 15 Å in the *z*-direction to prevent unrealistic interactions between neighboring images. Three layers of a total of nine atomic layers were allowed to have atomic relaxation under the same convergence tolerance as the bulk model. Monkhorst-Pack grid points with 4 × 4 × 1 *k*-meshes were utilized for optimizing the slab models. The final bismuth-rich BiVO_4_ (010) surface was developed through the optimization process. A variety of surface oxygen vacancy models were subsequently generated by removing an oxygen atom under the consideration of all potential configurations. Among these, the most stable bismuth-rich BiVO_4-x_ surface model was finally obtained by the optimization of all these configurations.

The adsorption energy (${E}_{a}$) of glycerol on a slab surface model can be defined as follows:

15 $${{{{{\rm{E}}}}}}_{{{{{\rm{a}}}}}}={{{{{\rm{E}}}}}}_{{{{{\rm{s}}}}}+{{{{\rm{glycerol}}}}}}-\left({{{{{\rm{E}}}}}}_{{{{{\rm{s}}}}}}+{{{{{\rm{E}}}}}}_{{{{{\rm{glycerol}}}}}}\right)$$

Here, ${E}_{s+{glycerol}}$, ${E}_{s}$, and ${E}_{{glycerol}}$ depict the DFT-calculated energies of a slab surface model with an adsorbed glycerol molecule, bare slab model, and glycerol molecule, respectively. Notably, a more negative adsorption energy indicates a stronger binding between the slab surface model and the glycerol molecule. The oxidation processes of glycerol on the bismuth-rich BiVO_4-x_ surface model were further explored to describe the free energy profiles associated with the reaction. Notably, the enthalpic and entropic contributions to the free energy were incorporated by the vibrational frequency calculations. Intermediate species predicted during the oxidation processes were fully considered, referring to relevant literature^14^.

### Supplementary information

Supplementary Information

Peer Review File

### Source data

Source Data

## Data Availability

Source data are provided with this paper.

## References

[1] Sheng H (2022). Linear paired electrochemical valorization of glycerol enabled by the electro-Fenton process using a stable NiSe_2_ cathode. Nat. Catal..

[2] Goetz MK, Bender MT, Choi KS (2022). Predictive control of selective secondary alcohol oxidation of glycerol on NiOOH. Nat. Commun..

[3] Luo L (2022). Selective photoelectrocatalytic glycerol oxidation to dihydroxyacetone via enhanced middle hydroxyl adsorption over a Bi_2_O_3_-incorporated catalyst. J. Am. Chem. Soc..

[4] An Z (2022). Pt_1_ enhanced C-H activation synergistic with Pt_n_ catalysis for glycerol cascade oxidation to glyceric acid. Nat. Commun..

[5] He Z (2022). Promoting biomass electrooxidation via modulating proton and oxygen anion deintercalation in hydroxide. Nat. Commun..

[6] Wang Y (2022). Efficient electrocatalytic oxidation of glycerol via promoted OH* generation over single-atom-bismuth-doped spinel Co_3_O_4_. ACS Catal..

[7] Han X (2020). Electrocatalytic oxidation of glycerol to formic acid by CuCo_2_O_4_ spinel oxide nanostructure catalysts. ACS Catal..

[8] Oh LS (2023). How to change the reaction chemistry on nonprecious metal oxide nanostructure materials for electrocatalytic oxidation of biomass-derived glycerol to renewable chemicals. Adv. Mater..

[9] Zhang Z, Huber GW (2018). Catalytic oxidation of carbohydrates into organic acids and furan chemicals. Chem. Soc. Rev..

[10] Kumar GS (2015). Stabilized glycerol dehydrogenase for the conversion of glycerol to dihydroxyacetone. Chem. Eng. J..

[11] Tang R (2021). Fabrication of MOFs’ derivatives assisted perovskite nanocrystal on TiO_2_ photoanode for photoelectrochemical glycerol oxidation with simultaneous hydrogen production. Appl. Catal. B Environ..

[12] Lu Y (2022). Boosting charge transport in BiVO_4_ photoanode for solar water oxidation. Adv. Mater..

[13] Gao R-T (2023). Dynamic semiconductor-electrolyte interface for sustainable solar water splitting over 600 h under neutral conditions. Sci. Adv..

[14] Liu D (2019). Selective photoelectrochemical oxidation of glycerol to high value-added dihydroxyacetone. Nat. Commun..

[15] Lin C (2023). Photo-electrochemical glycerol conversion over a mie scattering effect enhanced porous BiVO_4_ photoanode. Adv. Mater..

[16] Vo T-G, Kao C-C, Kuo J-L, Chiu C-c, Chiang C-Y (2020). Unveiling the crystallographic facet dependence of the photoelectrochemical glycerol oxidation on bismuth vanadate. Appl. Catal. B Environ..

[17] de Souza MBC (2019). Bi-modified Pt electrodes toward glycerol electrooxidation in alkaline solution: effects on activity and selectivity. ACS Catal..

[18] Feng S (2020). Experimental and theoretical investigation of the role of bismuth in promoting the selective oxidation of glycerol over supported Pt–Bi catalyst under mild conditions. ACS Catal..

[19] Kwon Y, Birdja Y, Spanos I, Rodriguez P, Koper MTM (2012). Highly selective electro-oxidation of glycerol to dihydroxyacetone on platinum in the presence of bismuth. ACS Catal..

[20] Cooper JK (2014). Electronic structure of monoclinic BiVO_4_. Chem. Mater..

[21] Jo WJ (2012). Phosphate doping into monoclinic BiVO_4_ for enhanced photoelectrochemical water oxidation activity. Angew. Chem. Int. Ed..

[22] Wang S, Chen P, Yun JH, Hu Y, Wang L (2017). An electrochemically treated BiVO_4_ photoanode for efficient photoelectrochemical water splitting. Angew. Chem. Int. Ed..

[23] Yu J, Kudo A (2006). Effects of structural variation on the photocatalytic performance of hydrothermally synthesized BiVO_4_. Adv. Funct. Mater..

[24] Lee D (2021). The impact of surface composition on the interfacial energetics and photoelectrochemical properties of BiVO_4_. Nat. Energy.

[25] Fitting Kourkoutis L, Hotta Y, Susaki T, Hwang HY, Muller DA (2006). Nanometer scale electronic reconstruction at the interface between LaVO_3_ and LaVO_4_. Phys. Rev. Lett..

[26] Tian Z (2019). Novel black BiVO_4_/TiO_2−x_ photoanode with enhanced photon absorption and charge separation for efficient and stable solar water splitting. Adv. Energy Mater..

[27] Jin B (2022). A two-photon tandem black phosphorus quantum dot-sensitized BiVO_4_ photoanode for solar water splitting. Energy Environ. Sci..

[28] Luo H (2008). Structural and photoelectrochemical properties of BiVO_4_ thin films. The J. Phys. Chem. C.

[29] Zhang K (2019). Black phosphorene as a hole extraction layer boosting solar water splitting of oxygen evolution catalysts. Nat. Commun..

[30] Wiktor J, Ambrosio F, Pasquarello A (2018). Role of polarons in water splitting: the case of BiVO_4_. ACS Energy Lett..

[31] Wu H (2022). Low-bias photoelectrochemical water splitting via mediating trap states and small polaron hopping. Nat. Commun..

[32] Gao RT (2023). Ru-P pair sites boost charge transport in hematite photoanodes for exceeding 1% efficient solar water splitting. Proc. Natl. Acad. Sci. USA..

[33] Zhang K (2020). Near-complete suppression of oxygen evolution for photoelectrochemical H_2_O oxidative H_2_O_2_ synthesis. J. Am. Chem. Soc..

[34] Lin C (2021). P-Type AsP nanosheet as an electron donor for stable solar broad-spectrum hydrogen evolution. ACS Appl. Mater. Inter..

[35] Yang W (2020). Benchmark performance of low-cost Sb2Se3 photocathodes for unassisted solar overall water splitting. Nat. Commun..

[36] Bu X, Wang G, Tian Y (2017). Foreign In^3+^ treatment improving the photoelectrochemical performance of a hematite nanosheet array for water splitting. Nanoscale.

[37] Chen H (2021). The role of surface states on reduced TiO_2_@BiVO_4_ photoanodes: enhanced water oxidation performance through improved charge transfer. ACS Catal..

[38] Trześniewski BJ (2017). Near-complete suppression of surface losses and total internal quantum efficiency in BiVO_4_ photoanodes. Energy Environ. Sci..

[39] Yang W, Prabhakar RR, Tan J, Tilley SD, Moon J (2019). Strategies for enhancing the photocurrent, photovoltage, and stability of photoelectrodes for photoelectrochemical water splitting. Chem. Soc. Rev..

[40] Klahr B, Gimenez S, Fabregat-Santiago F, Hamann T, Bisquert J (2012). Water oxidation at hematite photoelectrodes: the role of surface states. J. Am. Chem. Soc..

[41] Li H (2023). Boosting reactive oxygen species generation using inter-facet edge rich WO_3_ arrays for photoelectrochemical conversion. Angew. Chem. Int. Ed..

[42] Liu Y, Wang M, Zhang B, Yan D, Xiang X (2022). Mediating the oxidizing capability of surface-bound hydroxyl radicals produced by photoelectrochemical water oxidation to convert glycerol into dihydroxyacetone. ACS Catal..

[43] Hilbrands AM, Goetz MKK, Choi KS (2023). C–C Bond formation coupled with C–C bond cleavage during oxidative upgrading of glycerol on a nanoporous BiVO_4_ photoanode. J. Am. Chem. Soc..

[44] Wu Y-H, Kuznetsov DA, Pflug NC, Fedorov A, Müller CR (2021). Solar-driven valorisation of glycerol on BiVO_4_ photoanodes: effect of co-catalyst and reaction media on reaction selectivity. J. Mater. Chem. A.

[45] Luo L, Wang Z-j, Xiang X, Yan D, Ye J (2020). Selective activation of benzyl alcohol coupled with photoelectrochemical water oxidation via a radical relay strategy. ACS Catal..

[46] Liang S (2014). Monolayer HNb_3_O_8_ for selective photocatalytic oxidation of benzylic alcohols with visible light response. Angew. Chem. Int. Ed..

[47] Wan S (2022). Tuning the surface wettability of a BiVO_4_ photoanode for kinetically modulating water oxidative H_2_O_2_ accumulation. ACS Energy Lett..

[48] Barbieriková Z, Dvoranová D, Brezová V (2018). Photoinduced transformation of glycerol in titania suspensions.(An EPR spin trapping study of radical intermediates). Catal. Today.

[49] Zhang L (2022). Diketone-mediated photochemical reduction of selenite to elemental selenium: Role of carbon-centered radicals and complexation. Chem. Eng. J..

[50] Zhao Y (2021). α-Fe_2_O_3_ as a versatile and efficient oxygen atom transfer catalyst in combination with H_2_O as the oxygen source. Nat. Catal..

[51] Li J (2021). Reaction kinetics and interplay of two different surface states on hematite photoanodes for water oxidation. Nat. Commun..

[52] Ran L (2022). Engineering single-atom active sites on covalent organic frameworks for boosting CO_2_ photoreduction. J. Am. Chem. Soc..

[53] Wang L (2021). Suppressing water dissociation via control of intrinsic oxygen defects for awakening solar H_2_O-to-H_2_O_2_ generation. Small.

[54] Fan X (2024). Boosting bulk charge transport of CuWO_4_ photoanodes via Cs doping for solar water oxidation. Chin. J. Struct. Chem..

[55] Lin C (2023). Covalent organic frameworks bearing Ni active sites for free radical-mediated photoelectrochemical organic transformations. Sci. Adv..

[56] Tang D (2024). A controlled non-radical chlorine activation pathway on hematite photoanodes for efficient oxidative chlorination reactions. Chem. Sci..

[57] Kresse G (1996). Efficiency of ab-initio total energy calculations for metals and semiconductors using a plane-wave basis set. Comput. Mater. Sci..

[58] Kresse G (1996). Efficient iterative schemes for ab initio total-energy calculations using a plane-wave basis set. Phys. Rev. B.

[59] Blöchl PE (1994). Projector augmented-wave method. Phys. Rev. B Condens. Matter.

[60] Perdew JP (1996). Generalized gradient approximation made simple. Phys. Rev. Lett..

[61] Kim TW (2015). Simultaneous enhancements in photon absorption and charge transport of bismuth vanadate photoanodes for solar water splitting. Nat. Commun.

[62] Monkhorst HJ (1976). Special points for Brillouin-zone integrations. Phys. Rev. B Condens. Matter.

