# Supplementary material for: Solar-driven highly selective conversion of glycerol to dihydroxyacetone using surface atom engineered BiVO4 photoanodes
Source: Nat Commun. 2024 Jun 28;15:5475. doi: 10.1038/s41467-024-49662-7 (PMC11213950; doi:10.1038/s41467-024-49662-7)
Supplement: Supplementary file 1 — Supplementary Information [file 41467_2024_49662_MOESM1_ESM.pdf]

Supplementary Information:

Solar-Driven Highly Selective Conversion of Glycerol to Dihydroxyacetone Using Surface Atom Engineered $BiVO_4$ Photoanodes

Y. Lu et al.

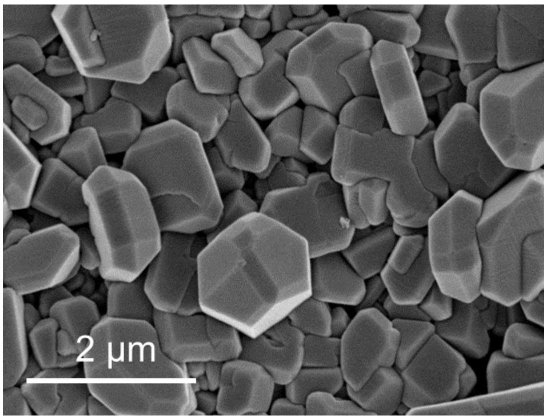

Supplementary Fig. 1. SEM top-view of $BiVO_4$ photoanode.

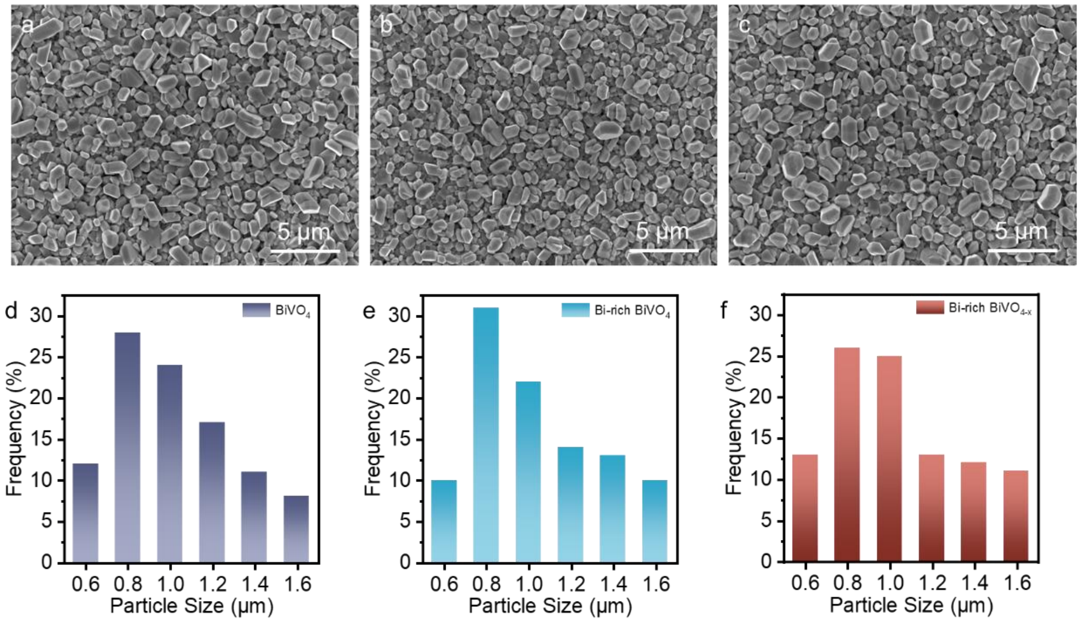

Supplementary Fig. 2. a-c) SEM images and d-f) particle size distribution of $BiVO_4$, Bi-rich $BiVO_4$, and Bi-rich $BiVO_{4-x}$ photoanodes at lower magnifications.

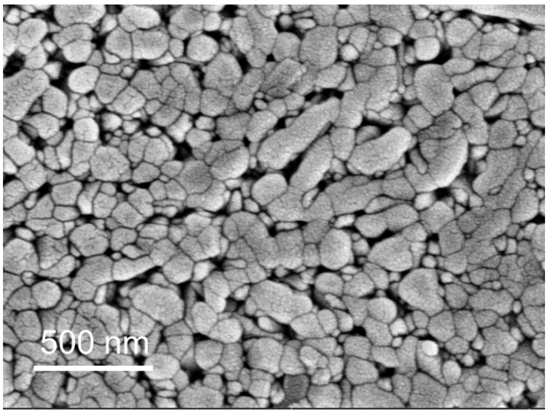

Supplementary Fig. 3. SEM top-view of $BiVO_4$ seed layer.

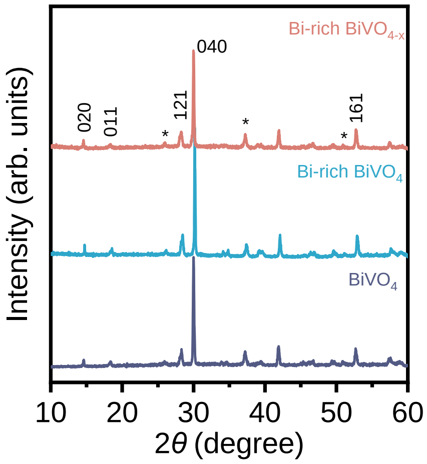

Supplementary Fig. 4. XRD patterns of $BiVO_4$, Bi-rich $BiVO_4$ and Bi-rich $BiVO_{4-x}$ photoanodes.

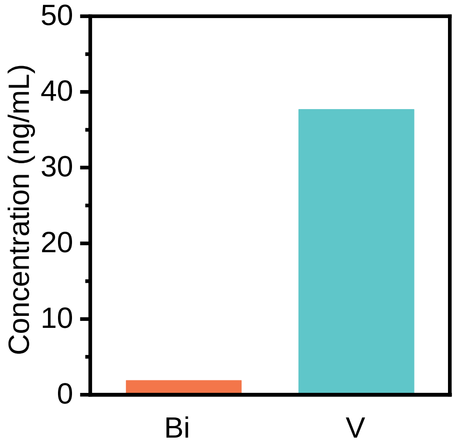

Supplementary Fig. 5. The ICP-MS spectrometry of NaOH solution after soaking $BiVO_4$.

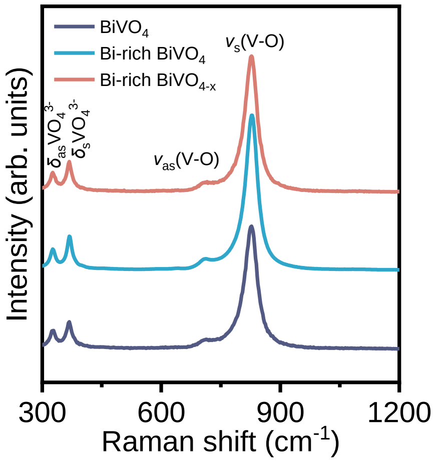

Supplementary Fig. 6. Raman spectra of $BiVO_4$, Bi-rich $BiVO_4$ and Bi-rich $BiVO_{4-x}$ photoanodes.

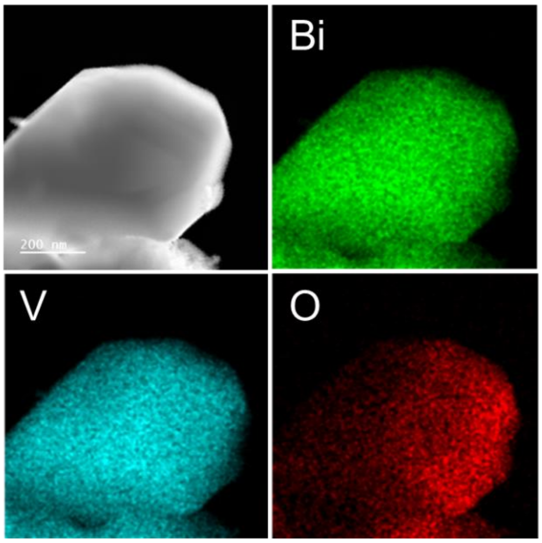

Supplementary Fig. 7. HAADF-STEM-EDX element mapping of Bi-rich $BiVO_{4-x}$.

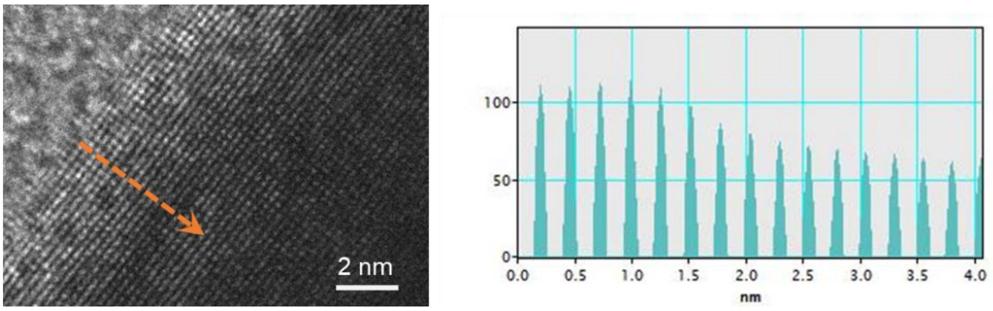

Supplementary Fig. 8. Enlarged HRTEM images of Bi-rich $BiVO_{4-x}$, and the inverse fast Fourier transform (IFFT) of surface to bulk.

Since the V atoms at the surface are precipitated, the Bi atoms with larger size tend to be directly exposed, so a stronger IFFT signal appears near the surface.

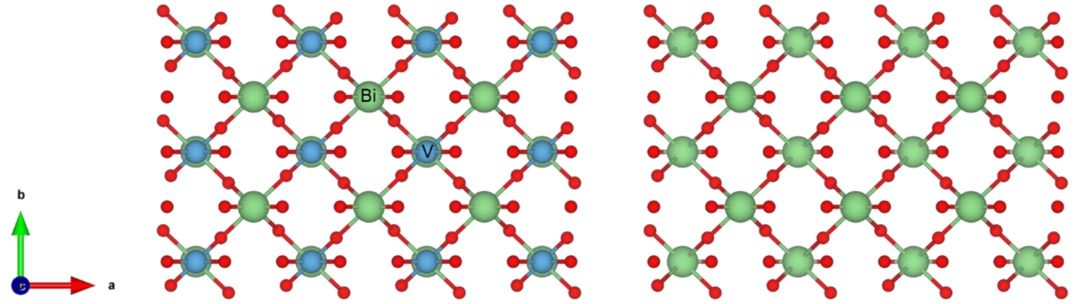

Supplementary Fig. 9. The Schematic diagrams of original $BiVO_4$ surface (left) and Bi-rich surface (right).

As shown above, the surface of pure $BiVO_4$ tends to alternate between Bi and V atoms (left), a state in which the surface exposed Bi atoms are limited. By employing alkali leaching, the most surface-covered V atoms can be removed, leaving Bi-rich surface (right).

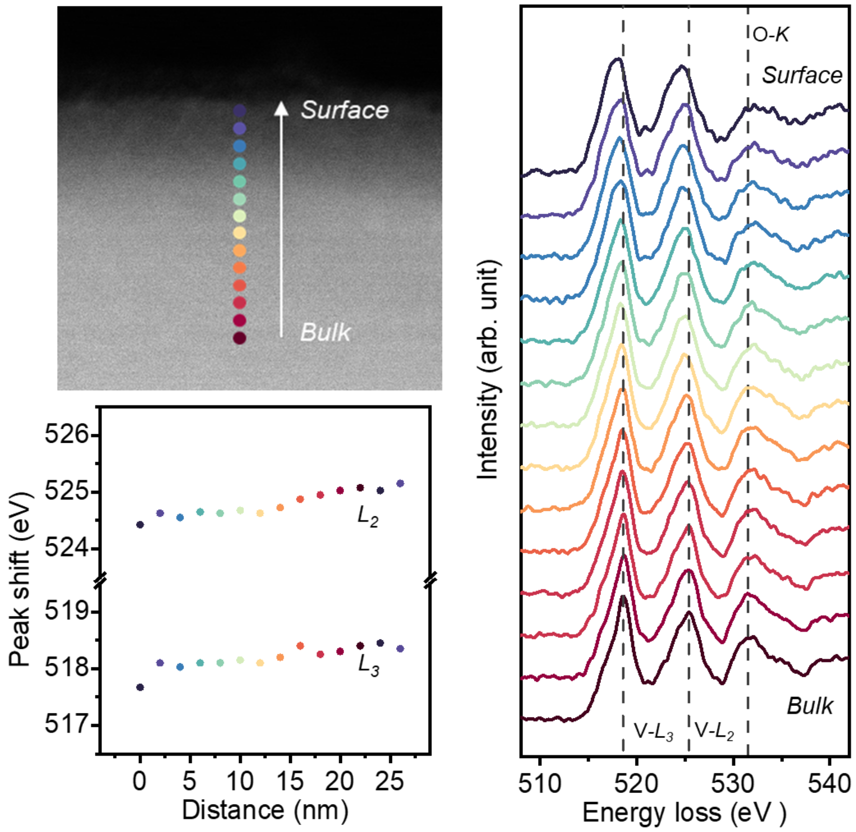

Supplementary Fig. 10. The corresponding EELS spectrum of $BiVO_4$, scale bar: 2 nm.

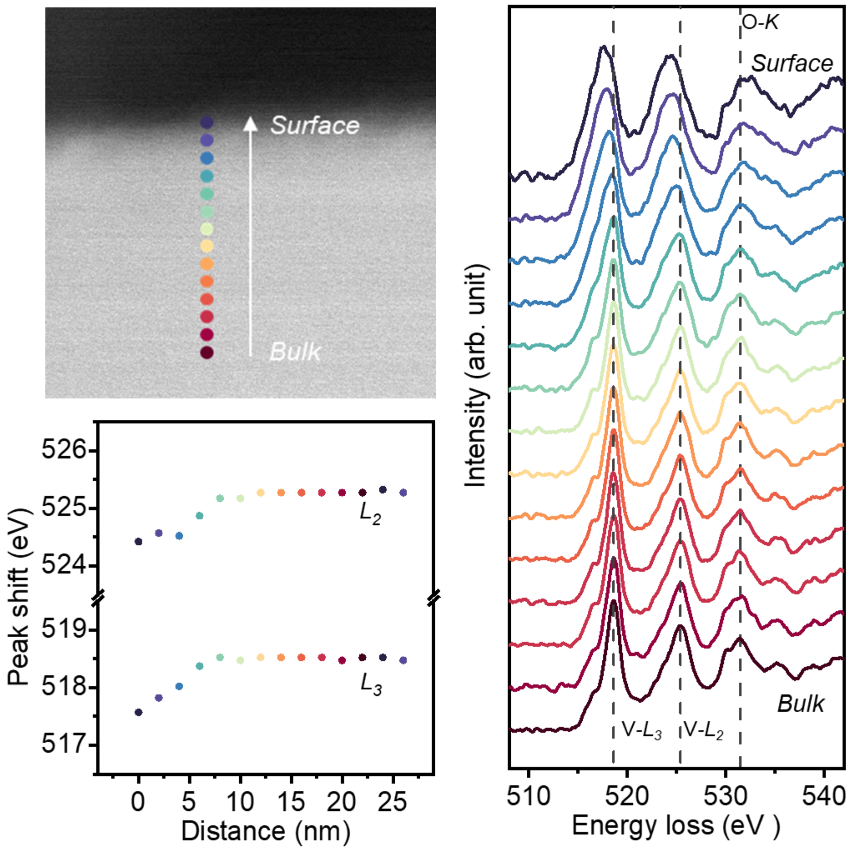

Supplementary Fig. 11. The corresponding EELS spectrum of Bi-rich $BiVO_4$, scale bar: 2 nm.

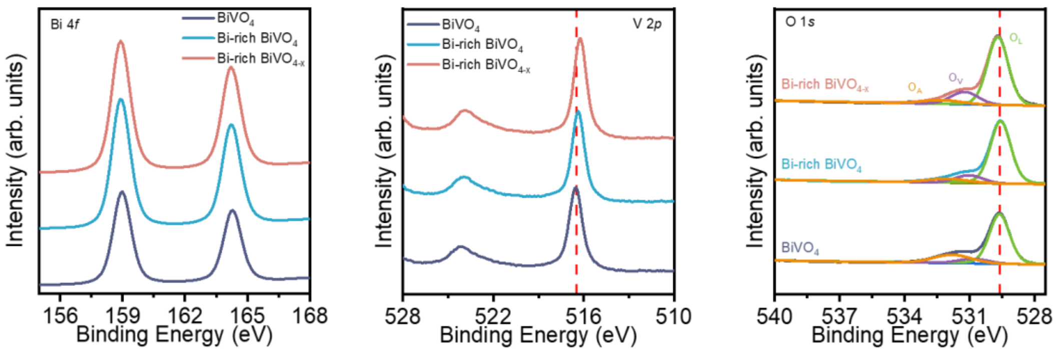

Supplementary Fig. 12. The XPS spectra of $BiVO_4$, Bi-rich $BiVO_4$ and Bi-rich $BiVO_{4-x}$ photoanodes.

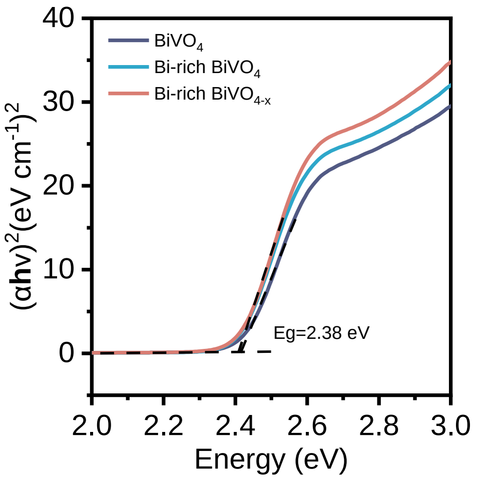

Supplementary Fig. 13. Tacu plots of the adsorption spectra of $BiVO_4$, Bi-rich $BiVO_4$ and Bi-rich $BiVO_{4-x}$ photoanodes.

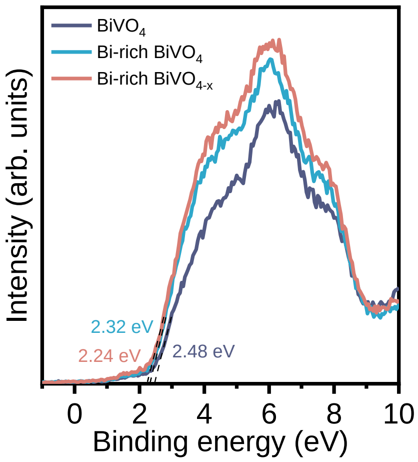

Supplementary Fig. 14. VB-XPS spectra of $BiVO_4$, Bi-rich $BiVO_4$ and Bi-rich $BiVO_{4-x}$ photoanodes.

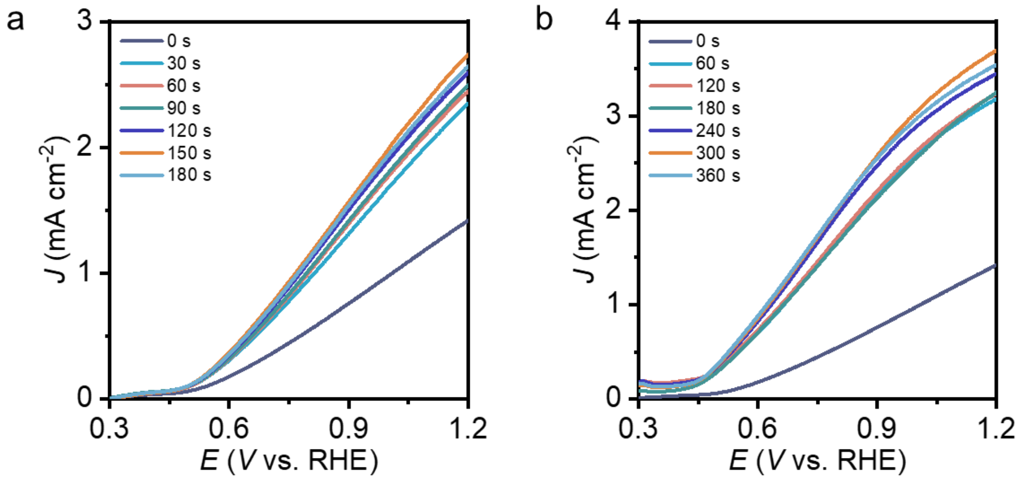

Supplementary Fig. 15. a) LSV curves of Bi-rich $BiVO_4$ obtained at different alkali soaking times. b) LSV curves of $BiVO_{4-x}$ obtained at electrochemical reduction times.

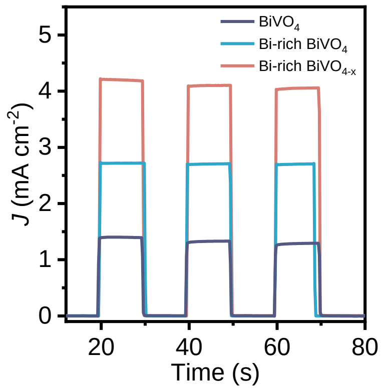

Supplementary Fig. 16. Chopped *J-V* curves of $BiVO_4$, Bi-rich $BiVO_4$ and Bi-rich $BiVO_{4-x}$ photoanodes at 1.23 V_{RHE} in 0.5 M Na_2SO_4 at pH = 2 with glycerol under AM 1.5G illuminations.

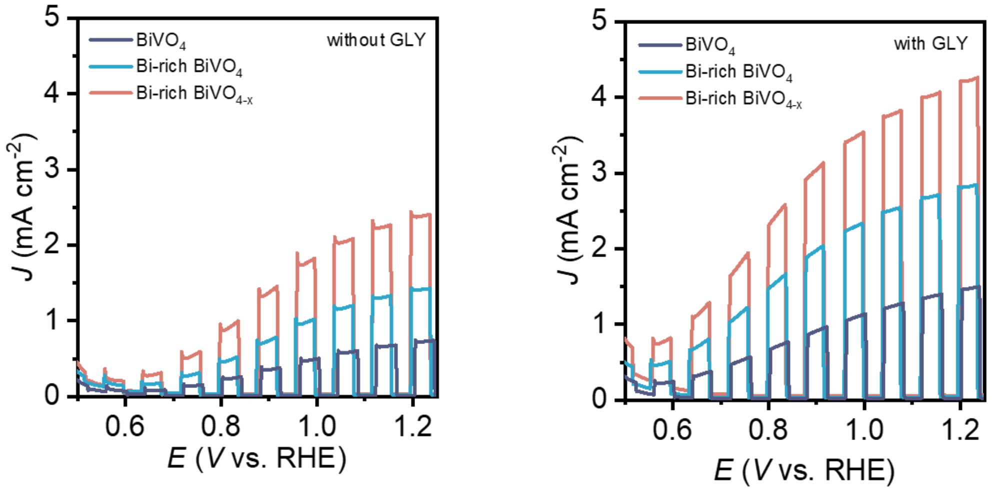

Supplementary Fig. 17. Chopped *J-V* curves of $BiVO_4$, Bi-rich $BiVO_4$ and Bi-rich $BiVO_{4-x}$ photoanodes in 0.5 M Na_2SO_4 at pH = 2 with/without glycerol under AM 1.5G illuminations.

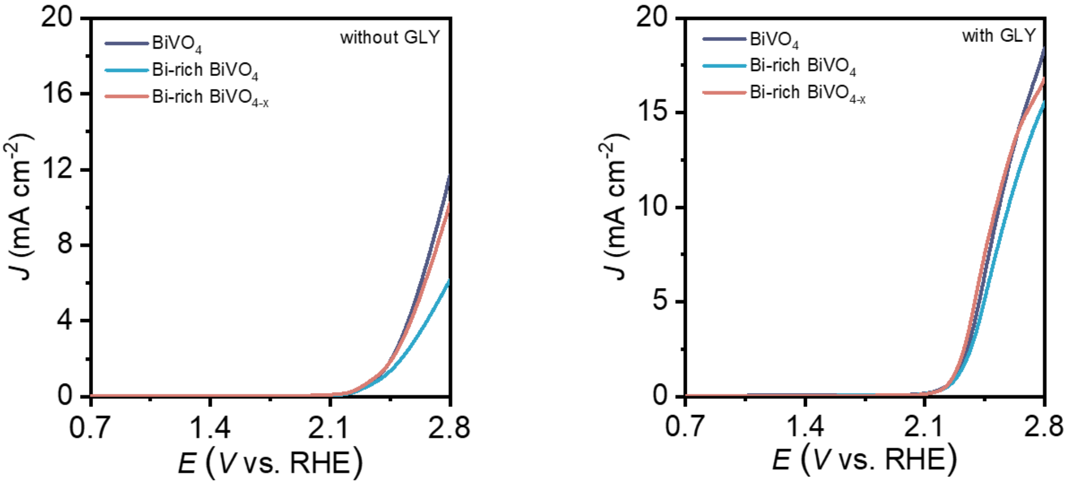

Supplementary Fig. 18. Dark *J-V* curves of $BiVO_4$, Bi-rich $BiVO_4$ and Bi-rich $BiVO_{4-x}$ photoanodes in 0.5 M Na_2SO_4 at pH = 2 with/without glycerol.

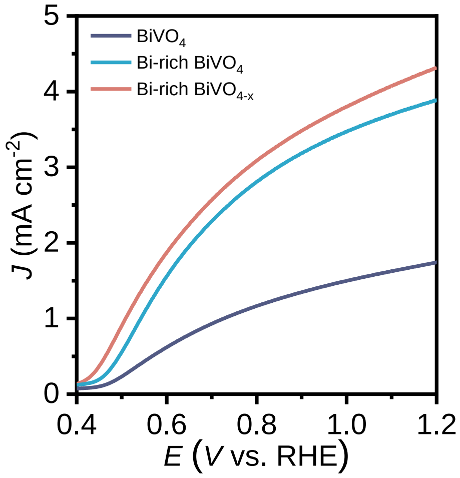

Supplementary Fig. 19. J-V curves of $BiVO_4$, Bi-rich $BiVO_4$ and Bi-rich $BiVO_{4-x}$ photoanodes measured in 0.5 M Na_2SO_4 containing 0.2 M Na_2SO_3 as hole scavenger under AM 1.5G illuminations.

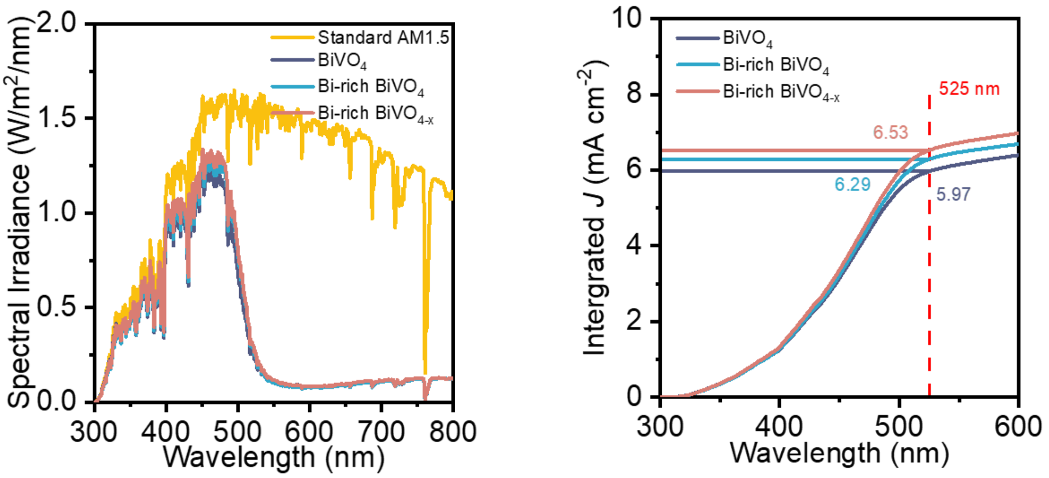

Supplementary Fig. 20. Spectra of the solar irradiance of AM 1.5G and corresponding J_{abs} that was calculated by the absorption spectra of $BiVO_4$, Bi-rich $BiVO_4$ and Bi-rich $BiVO_{4-x}$ photoanodes (300~525 nm).

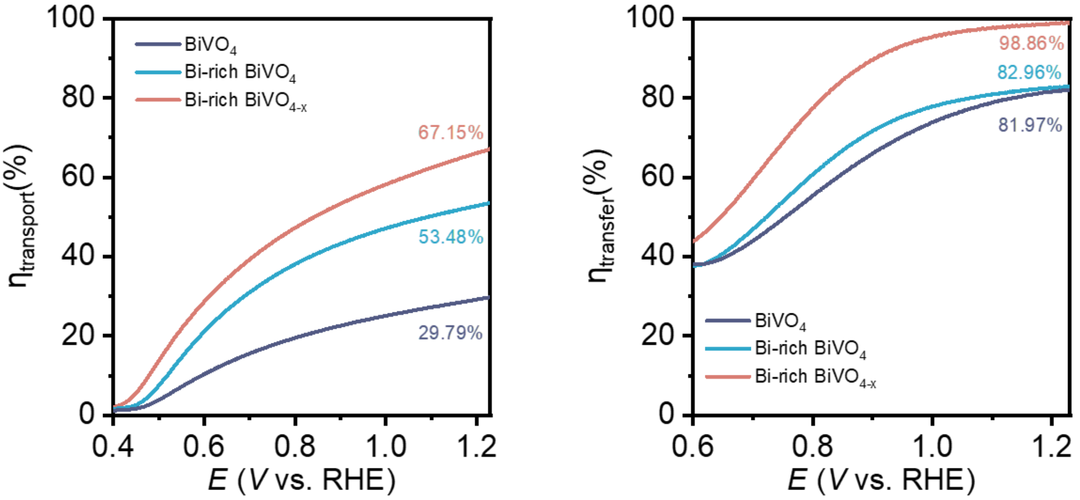

Supplementary Fig. 21. Calculated charge transport and transfer efficiencies of $BiVO_4$, Bi-rich $BiVO_4$ and Bi-rich $BiVO_{4-x}$ photoanodes.

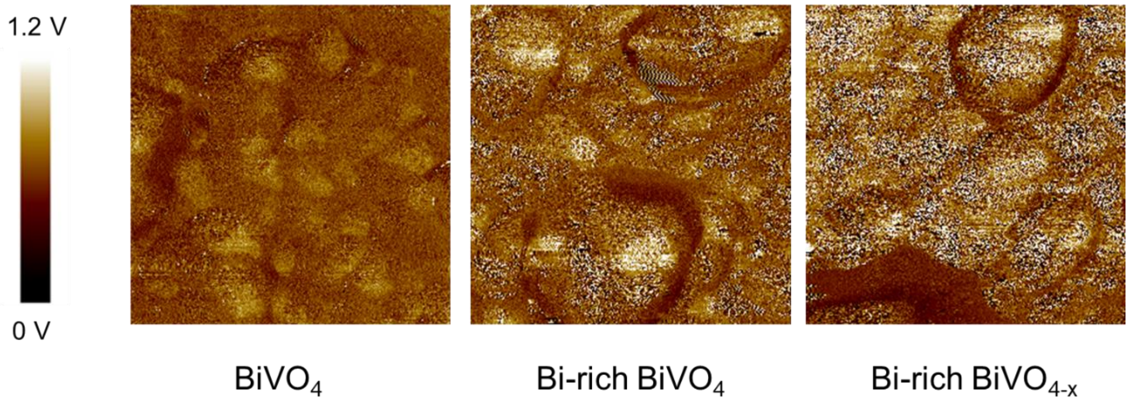

Supplementary Fig. 22. KPFM images of $BiVO_4$, Bi-rich $BiVO_4$ and Bi-rich $BiVO_{4-x}$ photoanodes.

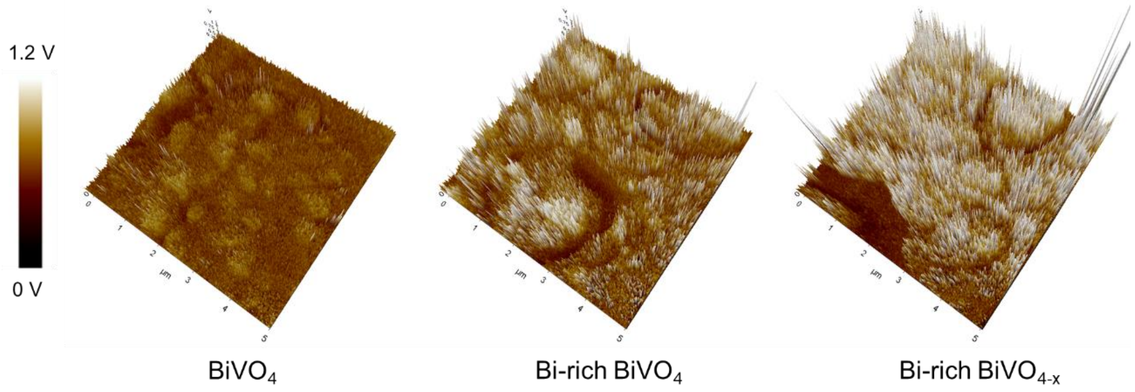

Supplementary Fig. 23. KPFM 3D-images of $BiVO_4$, Bi-rich $BiVO_4$ and Bi-rich $BiVO_{4-x}$ photoanodes.

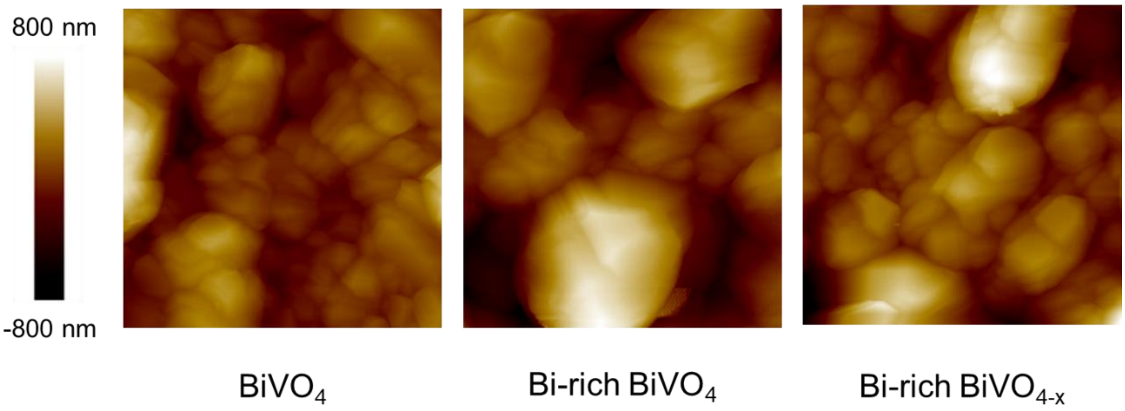

Supplementary Fig. 24. Topography of $BiVO_4$, Bi-rich $BiVO_4$ and Bi-rich $BiVO_{4-x}$ photoanodes.

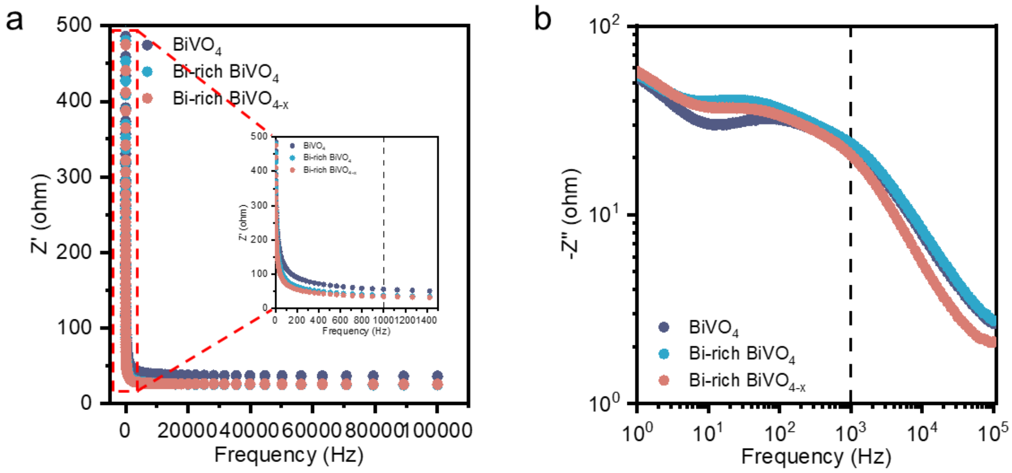

Supplementary Fig. 25. Bode plots of a) real and b) imaginary parts extracted from the EIS data for the $BiVO_4$, Bi-rich $BiVO_4$, and Bi-rich $BiVO_{4-x}$ photoanodes.

When the test frequency exceeds 1000 Hz, the trend aligns with the Mott-Schottky assumption, where the real part of the impedance is independent of frequency, and the imaginary part exhibits a log-log frequency dependence with a slope of -1.

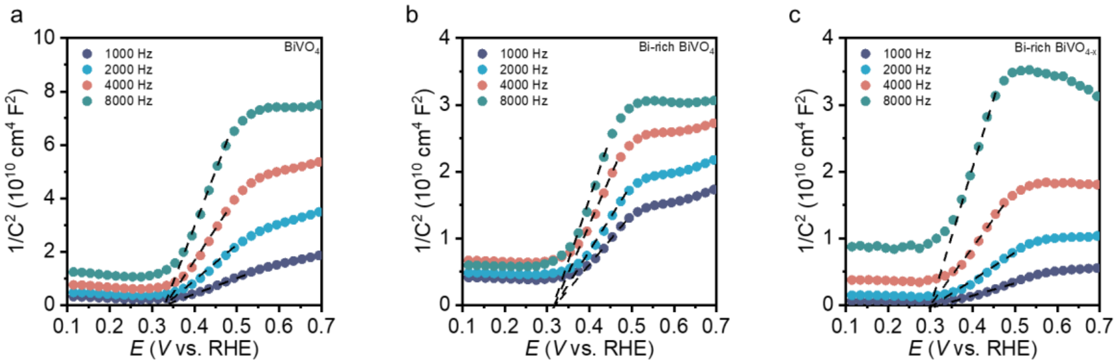

Supplementary Fig. 26. Varied frequency Mott−Schottky plots measured under AM 1.5 G illumination conditions for the a) $BiVO_4$, b) Bi-rich $BiVO_4$, and c) Bi-rich $BiVO_{4-x}$ photoanodes.

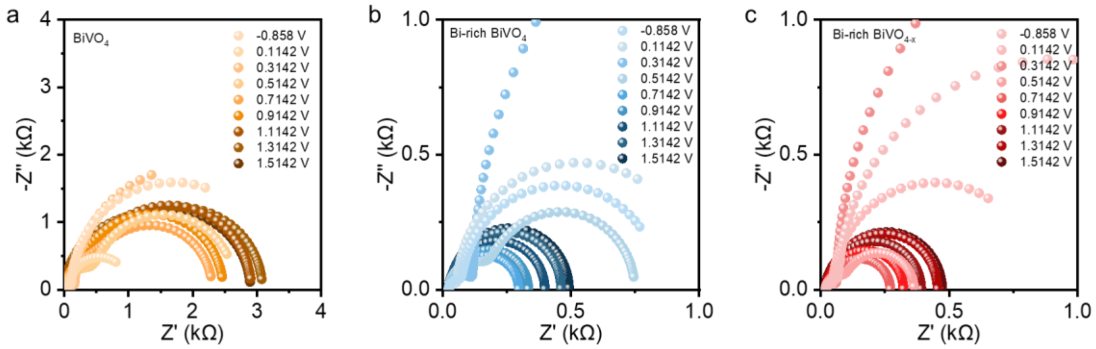

Supplementary Fig. 27. EIS data of a) $BiVO_4$, b) Bi-rich $BiVO_4$ and c) Bi-rich $BiVO_{4-x}$ photoanodes at different bias for PEC GOR under AM 1.5G illuminations.

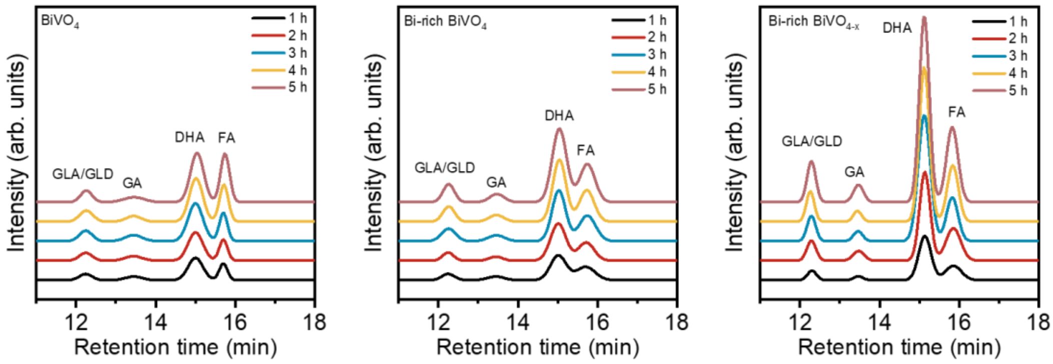

Supplementary Fig. 28. HPLC spectra of the products over $BiVO_4$, Bi-rich $BiVO_4$ and Bi-rich $BiVO_{4-x}$ photoanodes, collected once every hour of reaction.

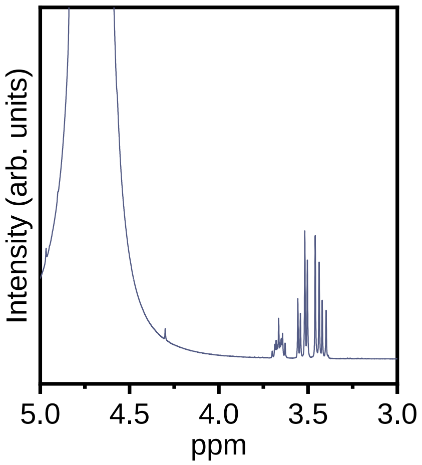

Supplementary Fig. 29. NMR spectra of glycerol oxidation products.

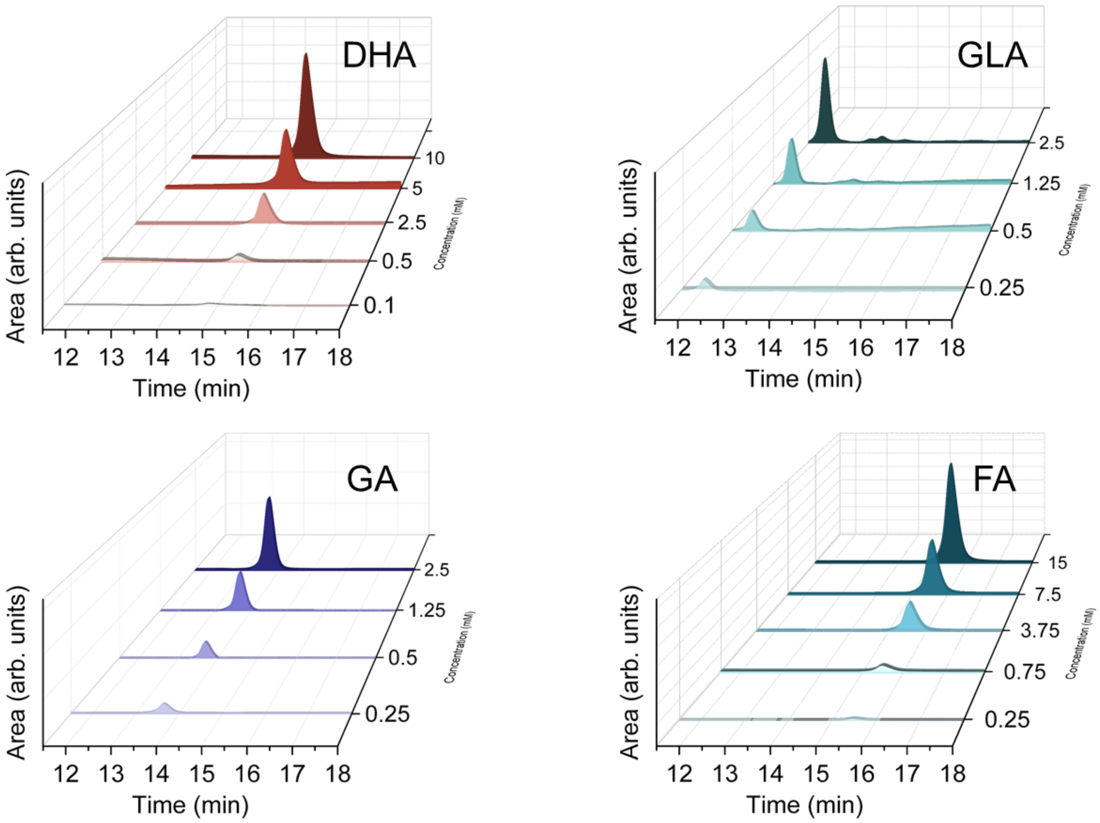

Supplementary Fig. 30. HPLC spectra of different concentrations of GOR products.

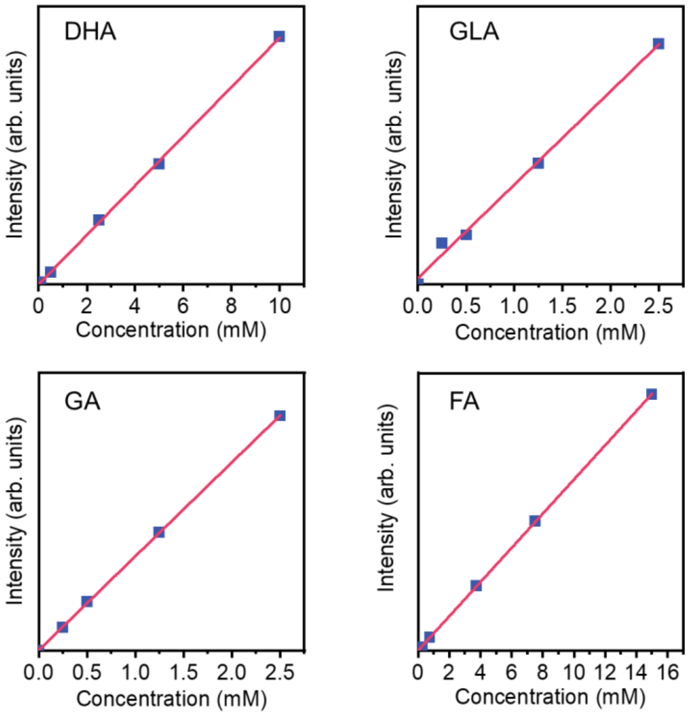

Supplementary Fig. 31. Concentration calibration curves for various GOR products.

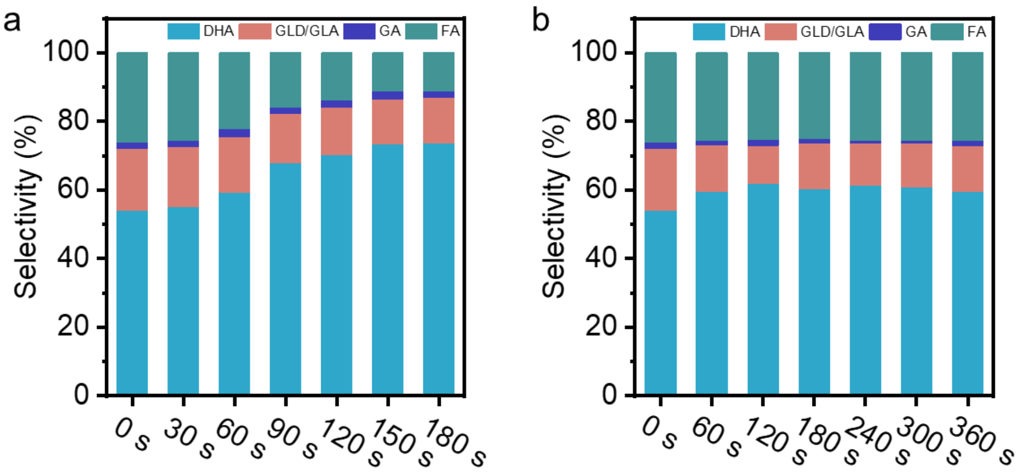

Supplementary Fig. 32. The GOR selectivity of a) Bi-rich $BiVO_4$ obtained at different alkali soaking times and b) $BiVO_{4-x}$ obtained at electrochemical reduction times.

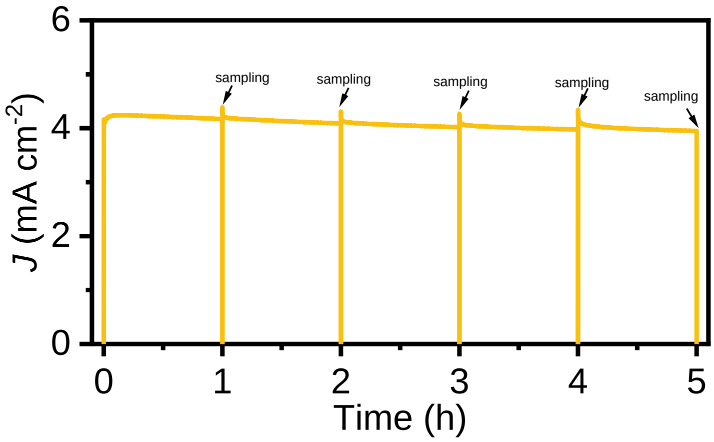

Supplementary Fig. 33. Long-term PEC GOR test over Bi-rich $BiVO_{4-x}$ photoanode, sampled each hour.

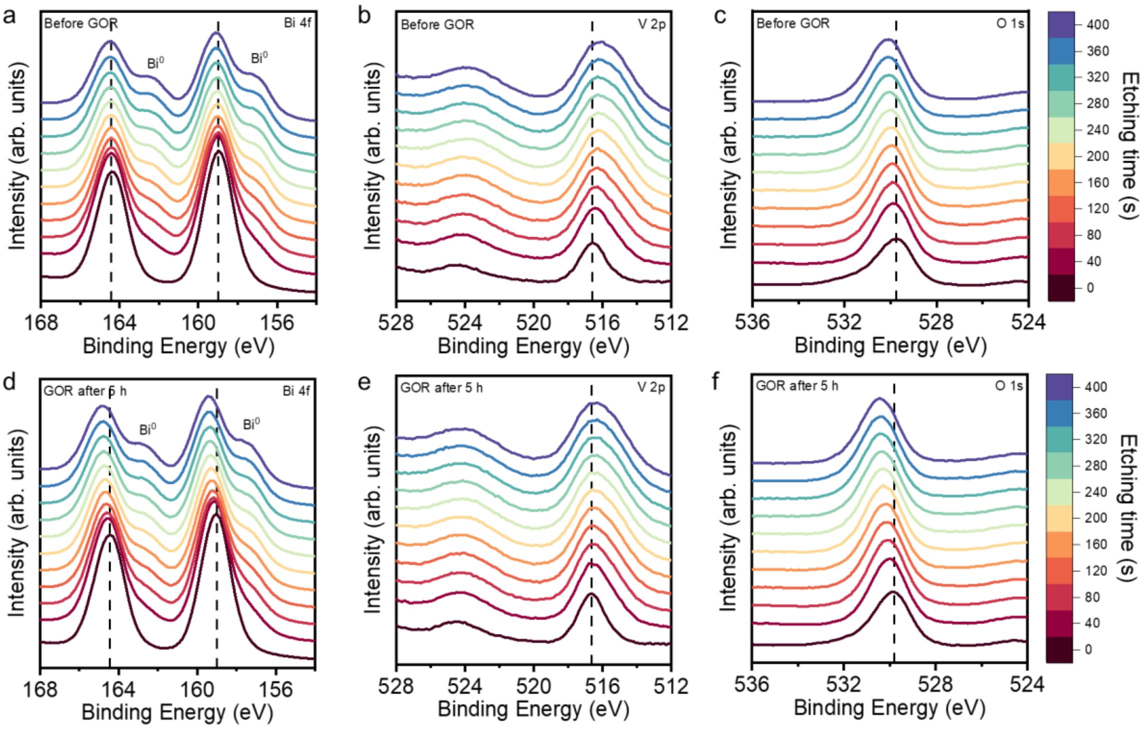

Supplementary Fig. 34. Etching XPS spectra of Bi-rich $BiVO_{4-x}$ photoanodes a-c) before GOR and d-f) after 5 h GOR. Etching was performed every 40 s for a total of 10 etchings. Under the effect of argon ion etching, part of Bi^{3+} was reduced to metallic Bi, so that the peaks of metallic Bi appeared at about 157.2 eV and 162.5 eV in the XPS spectra of Bi 4f.

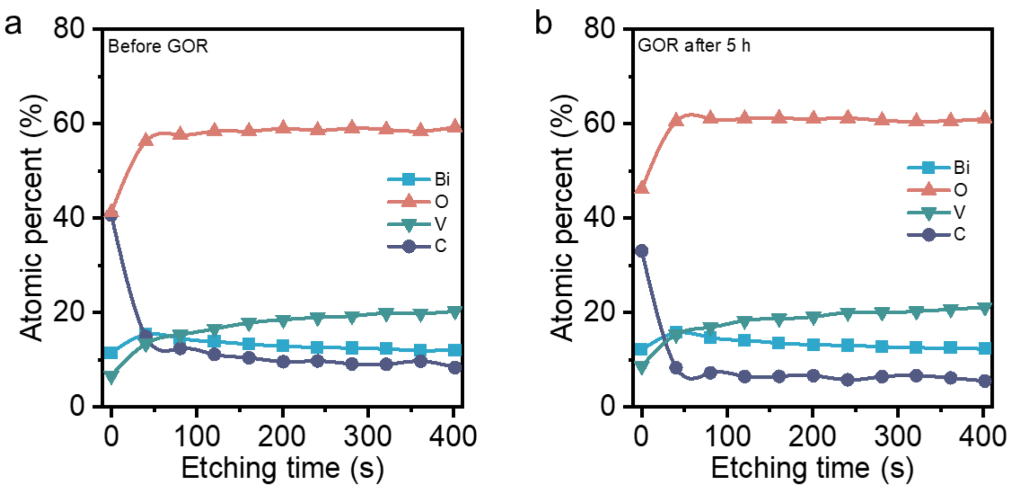

Supplementary Fig. 35. Summary of elemental percentages of Bi-rich $BiVO_{4-x}$ photoanodes a) before GOR and b) after 5 h GOR obtained from etching-XPS spectra. The element C present in this result is mainly from carbon contamination during XPS testing.

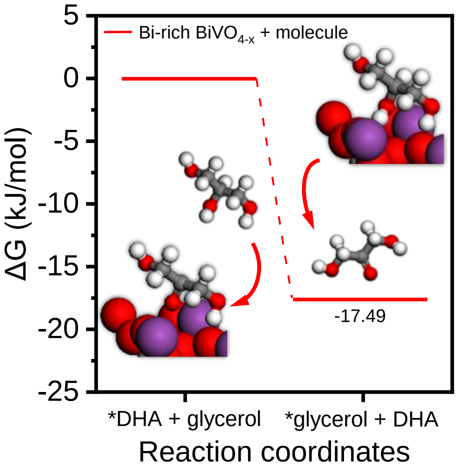

Supplementary Fig. 36. The change in the DFT-calculated Gibbs free energy associated with the approach of the glycerol (new reactant) toward the DHA-adsorbed Bi-rich $BiVO_{4-x}$ surface and subsequent release of the adsorbed DHA (product).

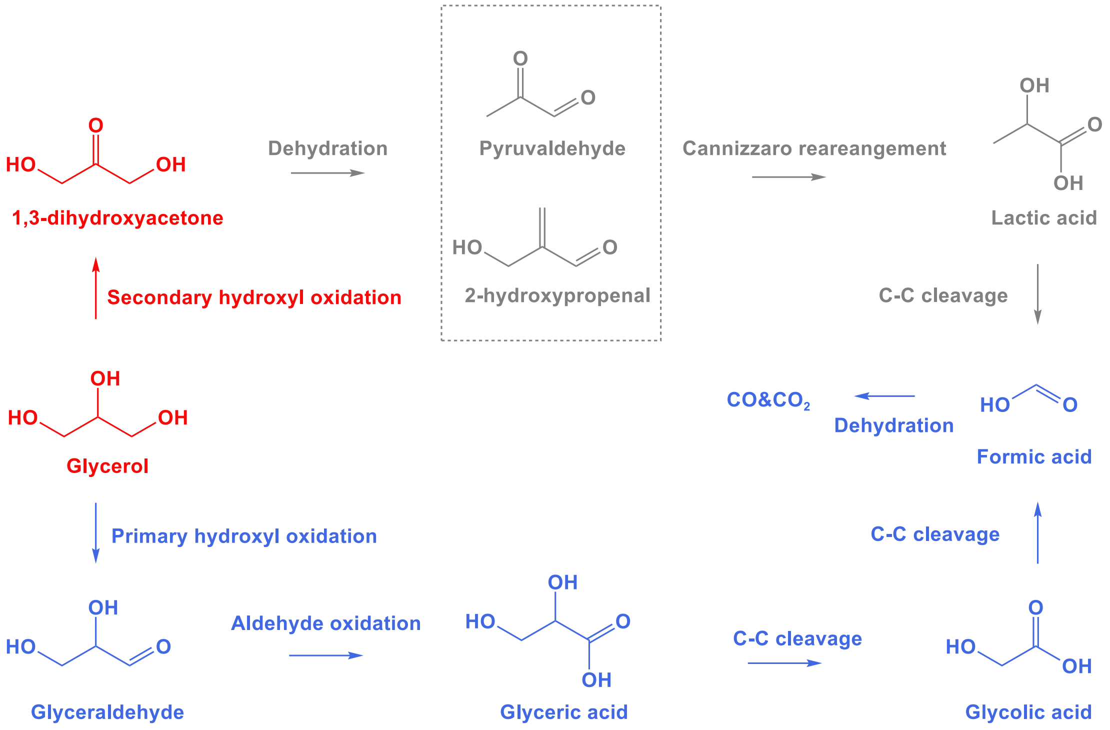

Supplementary Fig. 37. The reaction pathway of glycerol oxidation. The blue area is the primary hydroxyl oxidation reaction pathway, the red area is the secondary hydroxyl oxidation reaction pathway, and the gray area is the substances not detected by HPLC.

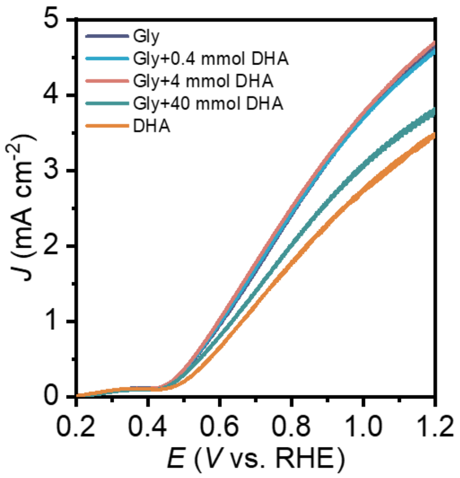

Supplementary Fig. 38 *J-V* curves of Bi-rich $BiVO_{4-x}$ photoanode in mixed solutions with different proportions of glycerol and DHA.

When the ratio of DHA to glycerol concentration reached 10:1, the mass transfer of glycerol was greatly limited, resulting in a significant inhibition of its oxidative behavior.

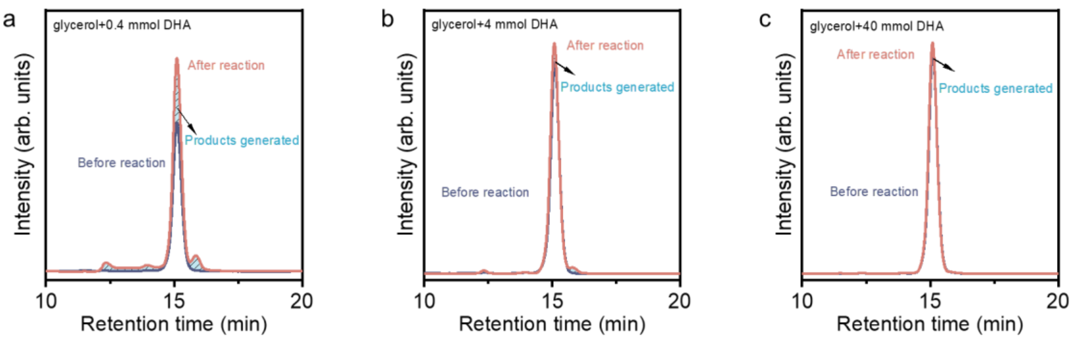

Supplementary Fig. 39 Comparison of HPLC spectra of Bi-rich $BiVO_{4-x}$ photoanode before and after 5 hours of reaction in mixed solutions of DHA and glycerol in a ratio of a) 1:10, b) 1:1 and c) 10:1.

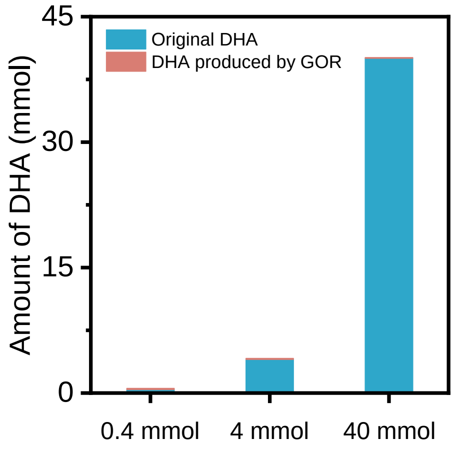

Supplementary Fig. 40. The amount of DHA in mixed solutions of glycerol and DHA in different proportions after being oxidized for 5 hours.

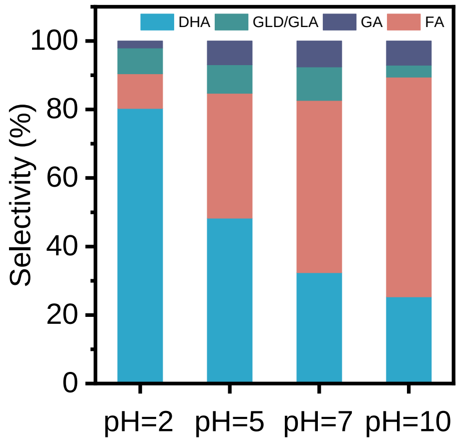

Supplementary Fig. 41. GOR selectivities of Bi-rich $BiVO_{4-x}$ photoanodes at different pH.

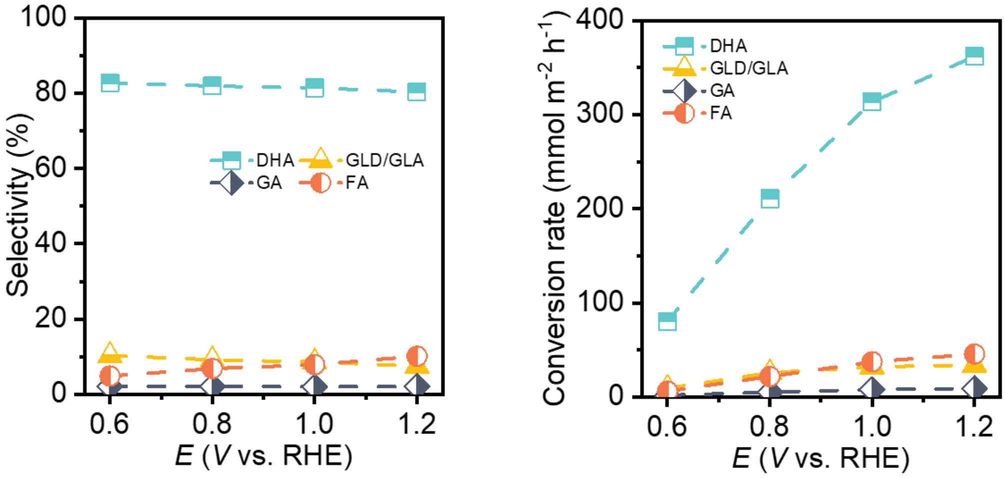

Supplementary Fig. 42. Conversion rate and selectivity of PEC glycerol oxidation products over Bi-rich $BiVO_{4-x}$ photoanodes at different potentials in 0.5 M Na_2SO_4 electrolyte.

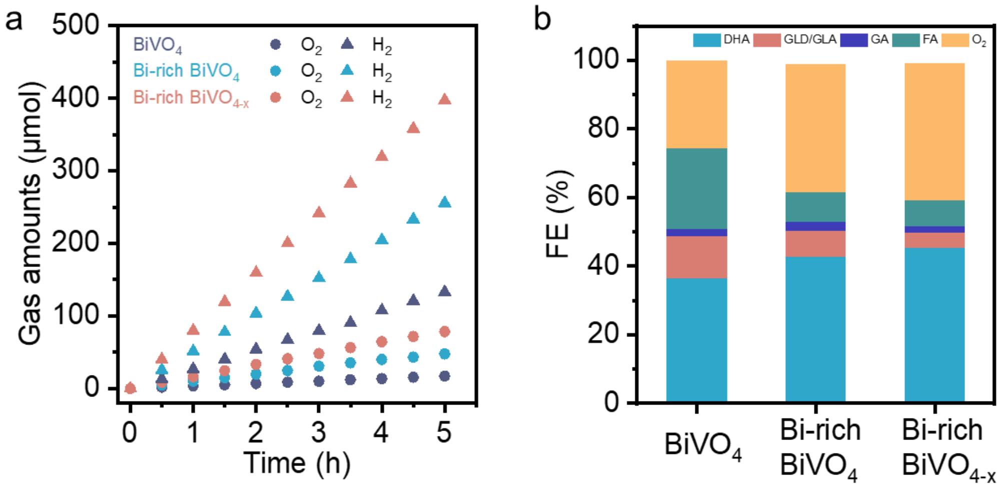

Supplementary Fig. 43. a) The actual quantities of H_2 and O_2 evolution in GOR under AM 1.5 illumination and b) total Faraday efficiency for all products.

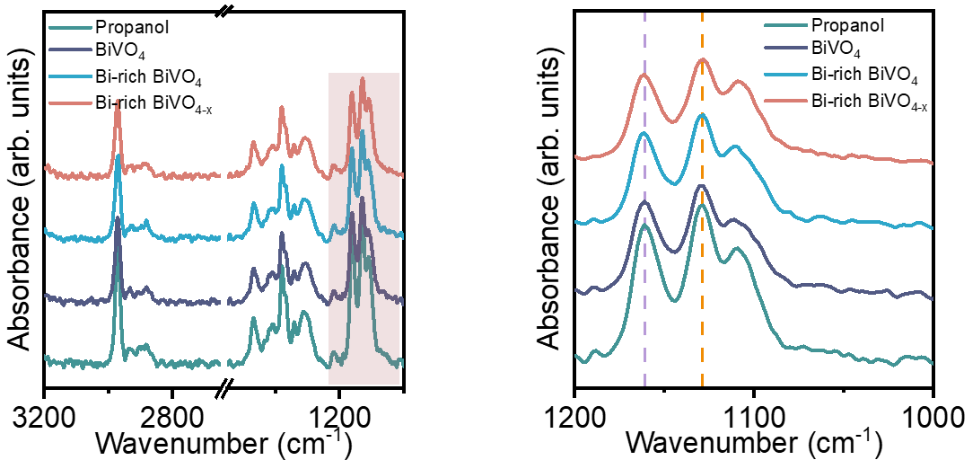

Supplementary Fig. 44. FT-IR spectra of propanol on $BiVO_4$, Bi-rich $BiVO_4$ and Bi-rich $BiVO_{4-x}$ photoanodes.

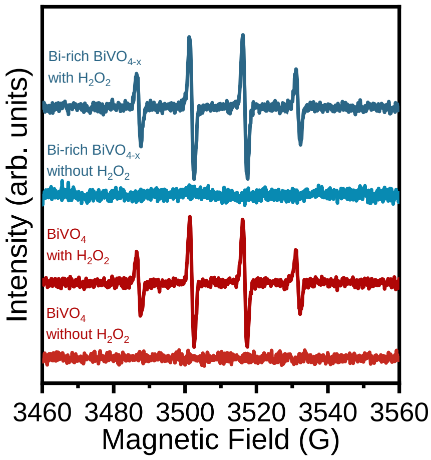

Supplementary Fig. 45. EPR detection of hydroxyl radicals over illuminated $BiVO_4$ and Bi-rich $BiVO_{4-x}$ photoanodes with/without H_2O_2.

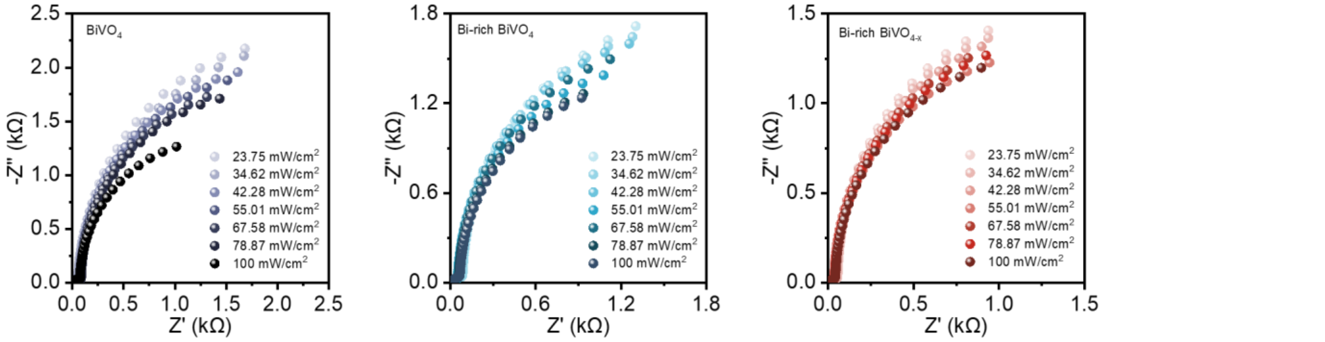

Supplementary Fig. 46. EIS data of $BiVO_4$, Bi-rich $BiVO_4$ and Bi-rich $BiVO_{4-x}$ photoanodes for glycerol oxidation. The impedance data were measured at the bias of 0.1142 V vs. RHE.

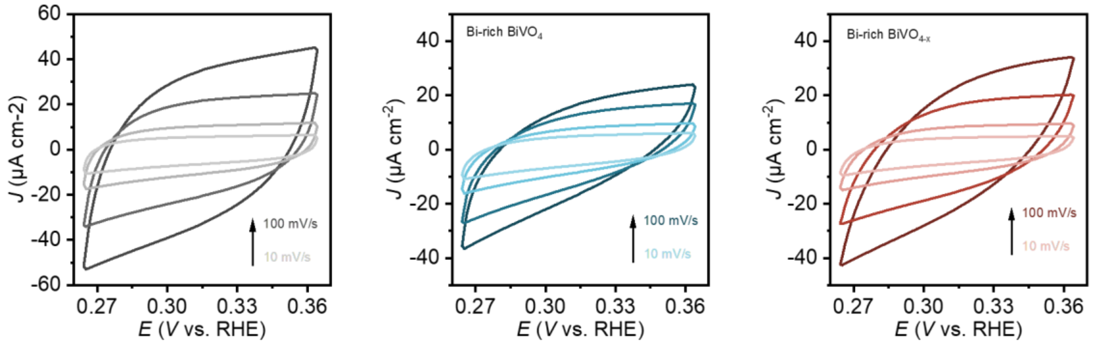

Supplementary Fig. 47. Cyclic voltammograms of $BiVO_4$, Bi-rich $BiVO_4$ and Bi-rich $BiVO_{4-x}$ photoanodes.

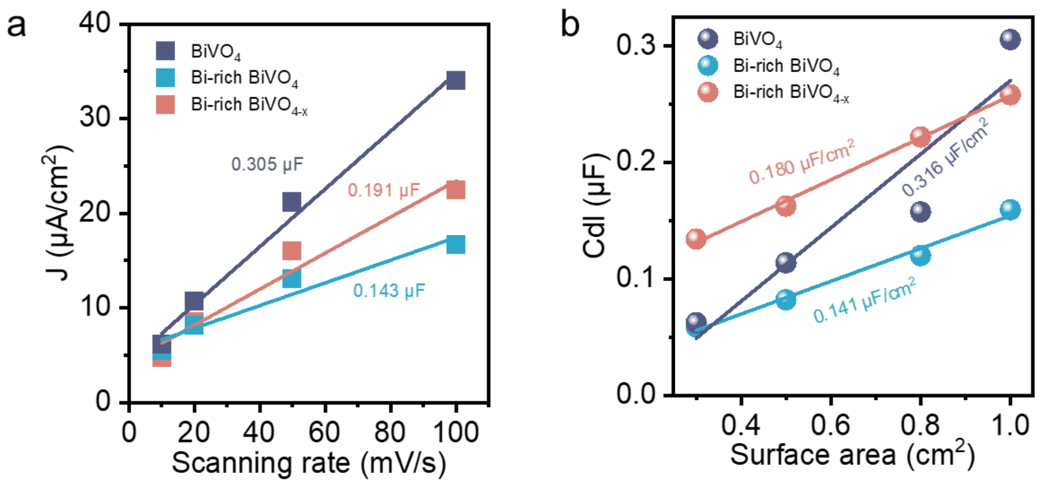

Supplementary Fig. 48. (a) Estimated double-layer capacitance (C_{dl}) and (b) specific C_{dl} for $BiVO_4$, Bi-rich $BiVO_4$ and Bi-rich $BiVO_{4-x}$ photoanodes.

The calculated ECSA for $BiVO_4$, Bi-rich $BiVO_4$ and Bi-rich $BiVO_{4-x}$ photoanodes are 0.97, 1.01 and 1.06 cm^2.

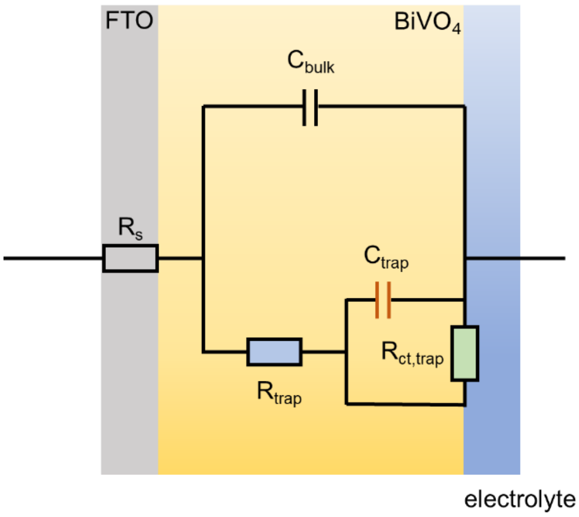

Supplementary Fig. 49. The equivalent model circuit for the fitting and simulation of EIS results.

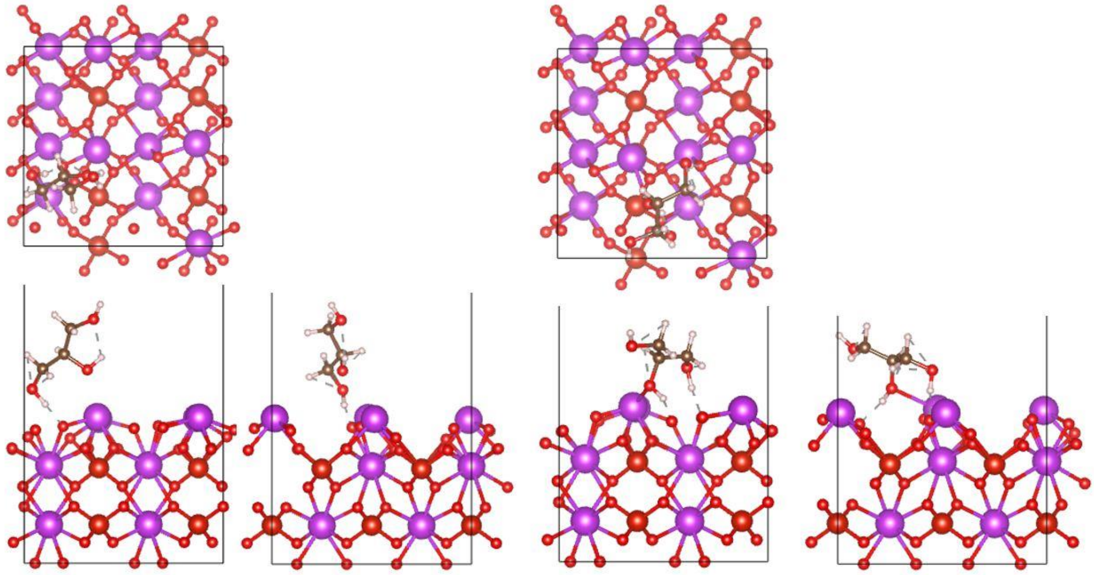

Supplementary Fig. 50. The adsorption model of primary and secondary hydroxyl groups of glycerol on the (010) crystal face of Bi-rich $BiVO_{4-x}$ photoanode.

[kJ/mol]	glycerol adsorbed on the surface	DHA in the empty box	DHA adsorbed on the surface	glycerol in the empty box	Preference dG
G	-46196.20647	-6460.44370	-45500.88844	-7138.27609	-17.48564

Supplementary Table S1. Specific values obtained from DHA desorption preference DFT calculations.

DHA desorption preference calculations were performed using the following equation:

dG = product - reactant = [(glycerol adsorbed on the surface) + (DHA in the empty box)] - [(DHA adsorbed on the surface) + (glycerol in the empty box)]

eU_{SHE}=0	DHA radical surface [eV]	DHA surface [eV]	1/2H_2 [eV]	eU_{SHE}=0 [eV]	ΔG [eV]	ΔG [kJ/mol]
G_0(pH=0)	-467.56762	-471.56819	-3.43200	0.00000	0.56857	54.86044
pH=2	-467.56762	-471.56819	-3.43200	0.11832	0.45025	43.44393
pH=14	-467.56762	-471.56819	-3.43200	0.82824	-0.25967	-25.05516

Supplementary Table S2. Specific data obtained from DFT calculations of DHA carbon chain dehydrogenation preferences in acidic and alkaline environments.

The specific calculation formula is shown below:

Alkaline:

$*DHA + OH^- \rightarrow *DHA \text{ based radical} + H_2O + e^-$

$\Delta G = \mu_{*DHA \text{ based radical}} - \mu_{*DHA} + \mu_{H2O} + \mu_{e-} - \mu_{OH-}$

(here, $\mu_{OH-} + \mu_{H+} = \mu_{H2O}$, $\mu_{OH-} - \mu_{e-} + \mu_{e-} + \mu_{H+} = \mu_{H2O}$)

$\mu_{e-} - \mu_{OH-} = \mu_{H+} + \mu_{e-} - \mu_{H2O}$

$\Delta G = \mu_{*DHA \text{ based radical}} - \mu_{*DHA} + \mu_{H2O} + \mu_{e-} - \mu_{OH-}$

$= \mu_{*DHA \text{ based radical}} - \mu_{*DHA} + \mu_{H2O} + \mu_{H+} + \mu_{e-} - \mu_{H2O}$

$= \mu_{*DHA \text{ based radical}} - \mu_{*DHA} + \mu_{H+} + \mu_{e-}$

$= \mu_{*DHA \text{ based radical}} - \mu_{*DHA} + 1/2\ \mu_{H2} - eU_{RHE}$

Acidic:

$*DHA \rightarrow *DHA \text{ based radical} + H^+ + e^-$

$\Delta G = \mu_{*DHA \text{ based radical}} - \mu_{*DHA} + \mu_{H+} + \mu_{e-}$

$= \mu_{*DHA \text{ based radical}} - \mu_{*DHA} + 1/2\ \mu_{H2} - eU_{RHE}$

$\mathbf{eU_{RHE}} = eU_{SHE} + k_BT * \ln 10 * pH = \mathbf{eU_{SHE} + 0.0592*pH}$ [eV] (T=300K)

Light intensity (mw/cm^2)	R_s (Ohm)	C_{bulk} ($\mu F/cm^2$)	R_{trap} (Ohm)	C_{trap} ($\mu F/cm^2$)	R_{ct} (Ohm)
23.75	22.54	31.56	88.64	323.52	4403
34.62	22.31	34.22	80.56	379.80	4224
42.28	22.58	37.86	72.51	464.99	4017
55.01	22.45	35.40	65.10	500.01	3893
67.58	22.61	41.62	64.76	531.87	3637
78.87	22.66	50.03	62.82	560.57	3506
100	22.01	72.45	60.70	639.07	2630

Supplementary Table S3. Fitted results of EIS curves for $BiVO_4$ photoanode at 0.1142 V vs. RHE under varied light intensity.

Light intensity (mw/cm^2)	R_s (Ohm)	C_{bulk} ($\mu F/cm^2$)	R_{trap} (Ohm)	C_{trap} ($\mu F/cm^2$)	R_{ct} (Ohm)
23.75	18.30	63.71	96.36	415.70	4056
34.62	18.56	74.55	82.67	476.83	3704
42.28	18.12	86.54	69.99	550.23	3318
55.01	18.29	111.38	59.07	592.61	3145
67.58	18.15	115.56	57.2	632.61	2890
78.87	18.26	137.58	48.34	677.30	2681
100	18.43	143.09	46.82	682.27	2620

Supplementary Table S4. Fitted results of EIS curves for Bi-rich $BiVO_4$ photoanode at 0.1142 V vs. RHE under varied light intensity.

Light intensity (mw/cm^2)	R_s (Ohm)	C_{bulk} ($\mu F/cm^2$)	R_{trap} (Ohm)	C_{trap} ($\mu F/cm^2$)	R_{ct} (Ohm)
23.75	15.45	91.47	75.66	460.77	3356
34.62	15.26	105.64	61.42	575.48	3024
42.28	15.03	114.26	48.50	670.60	2863
55.01	15.97	143.00	40.09	695.53	2771
67.58	15.99	150.97	35.57	703.12	2688
78.87	15.56	162.34	30.25	721.44	2561
100	15.69	168.57	28.33	726.51	2522

Supplementary Table S5. Fitted results of EIS curves for Bi-rich $BiVO_{4-x}$ photoanode at 0.1142 V vs. RHE under varied light intensity.

Reaction step	Reaction equation	Number of electrons transferred
1	$C_3H_8O_3 \rightarrow \cdot C_3H_7O_3 + H^+ + e^-$	1
2	$\cdot C_3H_7O_3 \rightarrow C_3H_6O_3 + H^+ + e^-$	1

Supplementary Table S6. Reaction process for the conversion of glycerol to DHA.

The process primarily consists of two steps: the initial oxidation of glycerol to a radical intermediate (1), and the further dehydrogenation and rearrangement of the radical intermediate to form DHA (2). Both processes involve a one-electron transfer, making the complete oxidation of glycerol to DHA involve the transfer of two electrons.
